# Non-linear relationship between serum cholesterol levels and cognitive change among older people in the preclinical and prodromal stages of dementia: a retrospective longitudinal study in Taiwan

**DOI:** 10.1186/s12877-024-05030-0

**Published:** 2024-05-30

**Authors:** Hsin-Te Chang, Po-Chi Chan, Pai-Yi Chiu

**Affiliations:** 1 Department of Psychology, College of Science, Chung Yuan Christian University, Taoyuan, Taiwan; 2 Research Assistant Center, Show Chwan Memorial Hospital, Changhua City, Changhua, Taiwan; 3 Department of Neurology, Show Chwan Memorial Hospital, Changhua City, Changhua, Taiwan; 4 Department of Applied Mathematics, College of Science, Tunghai University, Taichung, Taiwan

**Keywords:** Cholesterol, Cognitive function, Dementia, Mild cognitive impairment, Subjective cognitive decline

## Abstract

**Background:**

Adverse effects of rigorously lowering low-density lipoprotein cholesterol on cognition have been reported; therefore, we aimed to study the contribution of serum cholesterol in cognitive decline in older people with or without dementia.

**Methods:**

Cognitive function was assessed by the Cognitive Abilities Screening Instrument (CASI). We investigated associations between serum cholesterol with cognitive decline using multiple regressions controlling for the effects of demographics, vascular risk factors, and treatments.

**Results:**

Most associations between cholesterol and CASI scores could be explained by non-linear and inverted U-shaped relationships (*R*^*2*^ = 0.003–0.006, *p* < 0.016, Šidákcorrection). The relationships were most evident between changes in cholesterol and CASI scores in older people at the preclinical or prodromal stages of dementia *(R*^*2*^ = 0.02–0.064, *p* values < 0.016). There were no differences in level of changes in CASI scores between individuals in 1st decile and 10th decile groups of changes in cholesterol (*p* = 0.266–0.972). However, individuals in the 1st decile of triglyceride changes and with stable and normal cognitive functions showed significant improvement in CASI scores compared to those in the 10th decile (*t*(202) = 2.275, *p* values < 0.05).

**Conclusion:**

These findings could implicate that rigorously lowering cholesterol may not be suitable for the prevention of cognitive decline among older people, especially among individuals in preclinical or prodromal stages of dementia.

**Supplementary Information:**

The online version contains supplementary material available at 10.1186/s12877-024-05030-0.

## Introduction

Previous studies have suggested that midlife elevated serum total cholesterol (TC) may be associated with cognitive decline [1–5]. Previous studies revealed associations between high midlife TC and an increased risk of Alzheimer’s dementia (AD) or all-cause dementia [6]. However, there was no evidence supporting an association between all-cause dementia including AD and late-life TC [6–8].

Recent guidelines have suggested a rigorous management of cholesterol, especially low-density lipoprotein cholesterol (LDL-c), among people with cerebrovascular disease (CVD) risks [9–11]. However, there is no consensus on whether the rigorous management has protective effects on cognitive function [10–13]. In addition, optimal levels of TC, triglycerides (TG), and high-density lipoprotein (HDL) remain to be determined. Several recent studies have reported that relationship between circulating cholesterol and cognitive function may conform to an inverted U-shaped function [1, 14, 15].

Researchers have recently addressed importance of brain health in relationship between CVD risk and cognitive decline [16, 17], such that effects of serum cholesterol on cognitive function might be different in the course of dementia [5]. According to demyelination hypothesis of AD, reduction in integrity of axons in the brain may be an early sign of AD [18, 19]. Cholesterol is a major component of axons and cholesterol level has been reported to be associated with normal functioning and repairment of axons [20, 21]. In addition, recent studies have revealed the role of cholesterol in preventing the migration of tauopathies in the brain [22–25]. Thus, cholesterol might play a more important role in early stages of dementia as compared to its role in later stages. To the best of our knowledge, no studies have investigated the role of cognitive states among older people in relationship between serum cholesterol and cognitive function.

In this study, we longitudinally investigated individuals with subjective and/or objective cognitive decline as well as patients with dementia. We analyzed association between serum cholesterol and cognitive function at baseline and at approximately 2.5 years after the first examination. We hypothesized that (1) relationship between cholesterol and cognitive function was non-linear and inverted U-shaped, (2) drastic changes in serum cholesterol were associated with cognitive decline, and (3) relationship between serum cholesterol and cognitive function was different across individuals with different cognitive states.

## Methods

### Participants

**Fig. 1:**
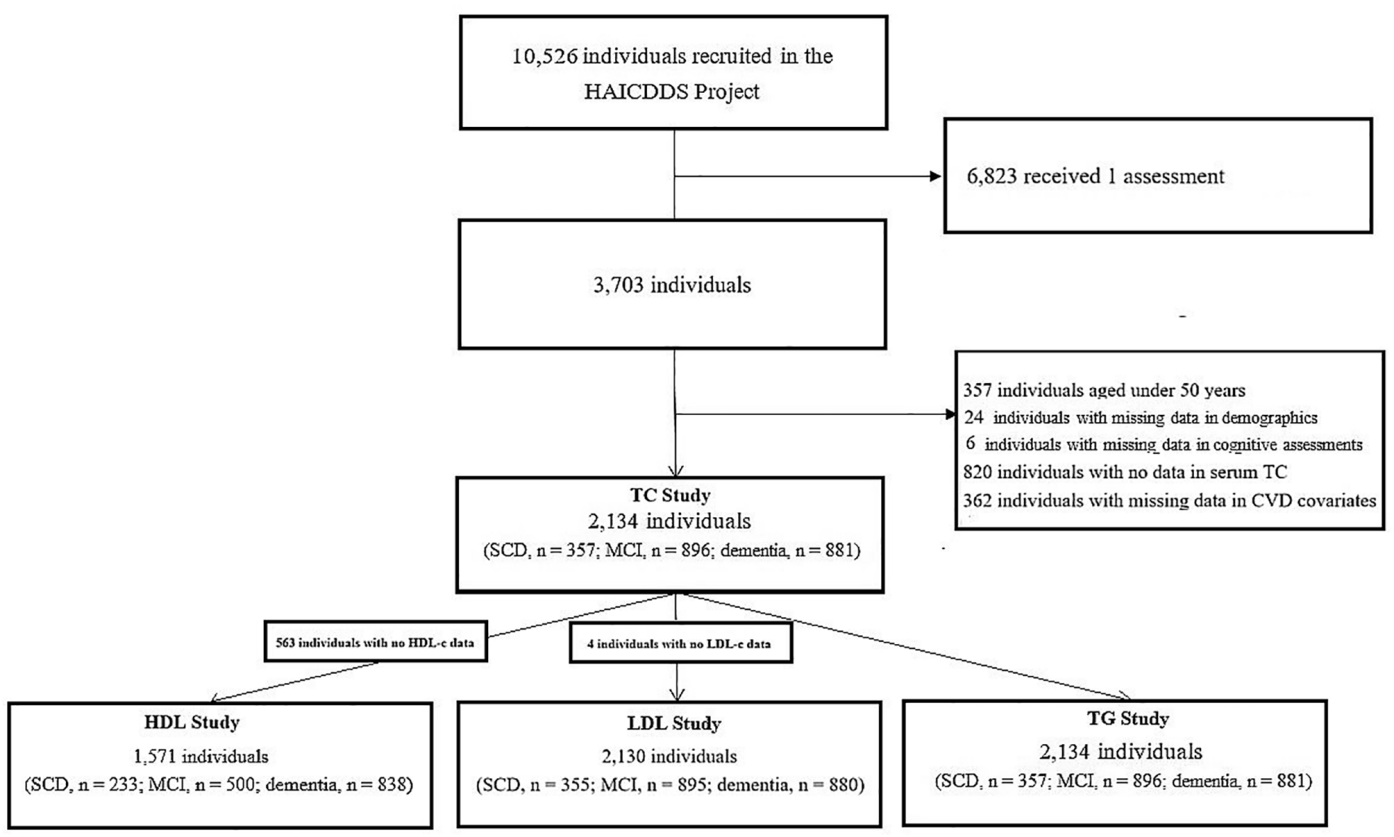
Selection process of study participants. CVD: cerebrovascular disease; HAICDDS, History-Based Artificial Intelligent Clinical Dementia Diagnostic System. CASI: Cognitive Assessment Screening Instrument; TC: Total cholesterol

The participants were selected from a dataset built for the “History-Based Artificial Intelligent Clinical Dementia Diagnostic System (HAICDDS) Project” [26]. HAICDDS aims to have participants longitudinally receive regular serum laboratory examinations and cognitive assessments at each time of evaluation at the Neurology outpatient clinics across Taiwan to facilitate early management of dementia. Individuals with no difficulties in understanding the instructions of cognitive assessment and blood sampling were included in the dataset. Currently, 10,526 participants have been included. Among them, 3,703 have been assessed at least twice. Data from 2,134 participants with at least two assessments in serum total cholesterol and cognitive function and aged over 50 years were extracted and analyzed in this study after excluding individuals aged under 50 years or with missing data in terms of demographics, serum total cholesterol levels, cerebrovascular diseases (CVD) and their risk factors, and drug treatment for the CVD (Fig. 1). The samples exclude from this study were younger (72.4 ± 13.7 years), more educated (6.5 ± 5.7 years), and more prone to be male (47%) as compared to the samples included in the analysis. In addition, they had higher cognitive (score on the Cognitive Abilities Screening Instrument [CASI]: 59.6 ± 30.3, maximum score = 100) and daily (score on the History-based Artificial Intelligent ADL questionnaire [HAI-ADL]: 9.9 ± 10.2, maximum score = 43) functions, lower levels of frailty (score on the Clinical Frailty Scale: 3.2 ± 2.7, maximum score = 7), and lower levels of vascular risks (hypertension: 44.6%; diabetes mellitus: 27.4%; coronary artery disease: 4.7%; arrhythmia: 6.6%) (absolute values of *t* = 3.73–14.71, *df* = 10,524; ${\chi }_{n=\text{10,526}, df=1}^{2}$ = 23.91–1189.00; all *p* values < 0.001). No difference in levels psychiatric symptoms (*p* = 0.38) was observed between individuals included in or excluded from the analysis. Data concerning HDL-c and LDL-c were available among 1,571 (73.6%) and 2,130 (99.9%) individuals, respectively, due to staff error. Neuroimaging studies were used to exclude marked etiologies other than degenerative or cerebrovascular. Participants who did not complete the assessment of cognitive functions were excluded. Data were analyzed retrospectively and anonymously. The study was approved by Committee for Medical Research Ethics of Show Chwan Memorial Hospital and the need for informed consent was waived (SCMH_IRB No: IRB1081006).

### Cognitive assessment

Global cognitive function among participants was assessed by clinical neuropsychologists using Cognitive Assessment Screening Instrument (CASI) [27]. CASI is a widely used tool assessing cognitive function with tasks evaluating memory, orientation, attention and concentration, language abilities, abstract thinking and judgment, and visuospatial abilities. Activities of daily living (ADL) of participants were assessed using History-based Artificial Intelligent ADL questionnaire (HAI-ADL) [28]. For diagnosing subjective cognitive decline (SCD), the individual should have a global Clinical Dementia Rating (CDR) score of 0 or 0.5 and perform normally on CASI with normal score HAI-ADL (< 8.5) [28]. Mild cognitive impairment (MCI) was diagnosed according to criteria proposed by previous studies [29], and an operational determination of MCI was made if the individual displayed a performance on CASI that was below cutoff score adjusted for age and educational level, a performance on HAI-ADL that was within normal range, and a CDR score was 0.5 with a CDR-sum of boxes < 4.5 [30–32]. Dementia was diagnosed according to NIA-AA criteria [33], and an operational determination of impaired cognitive function or ADL was made if the individual displayed performances below cutoffs of CASI and HAI-ADL with a CDR score of $\ge$ 0.5.

Participants with subjective or MCI (SMCI) at baseline assessment were further classified as following: (1) Individuals with incident dementia: Having SMCI at baseline assessment, displaying deterioration in ADL at follow-up assessment (i.e., from CDR = 0 or 0.5 to higher than or equal to 1), and without ‘reverse’ in cognitive function (i.e., from below the cutoff score to above or equal to the cutoff score) or ADL at any follow-up assessment (SMCI-D); or (2) Individuals without incident dementia (SMCI-stable, SMCI-S): Having SMCI at baseline assessment and without deterioration in ADL at follow-up assessment. Similarly, patients with dementia at baseline assessment were classified as following: (1) Patients with a functional deterioration (Dementia-D): Having prevalent dementia at baseline assessment and displaying a deterioration in ADL at follow-up assessment (i.e., from CDR = 1 to higher than or equal to 2); or (2) Patients without functional deterioration (Dementia-S): Having prevalent dementia at baseline assessment without a deterioration in ADL at follow-up assessment.

### Serum cholesterol

Serum TC and TG were directly measured using enzymatic methods. HDL-c was measured by the Roche HDL-c 3rd generation direct method. LDL-c was calculated by formula of Friedewald: LDL-c = TC – HDL-c – TG/5.0 (mg/dL) [34]. For individuals with TG $\ge$ 400 mg/dL, a revised Friedwald formula was used to better estimate LDL-c: LDL-c = TC – HDL-c – TG/8.0 (mg/dL) [35]. History of hypertension, diabetes mellitus, coronary artery disease, arrhythmia, and hypercholesterolemia as well as whether the patients were taking anti-hypertensives, anti-diabetics, and statins were collected through a structural interview or a review of medical charts.

### Statistical analysis

Statistical analyses were accomplished using SPSS 22.0 (IBM Corp., Armonk, NY) and R [36]. Longitudinal changes in serum TC, TG, HDL-c, and LDL-c levels as well as CASI scores were calculated as average in the difference in each pair of proximate follow-up examinations.

We established three models using multiple regressions that controlled for covariates (i.e., age, educational levels, sex, follow-up duration, hypertension, diabetes mellitus, coronary artery disease, cerebrovascular disease, arrhythmia, hypercholesterolemia, taking anti-hypertensive, taking anti-diabetics, taking anti-coagulants, taking statins, and frailty [37]) to investigate relationship between baseline cholesterol levels and CASI score (model 1), between baseline cholesterol levels and longitudinal changes in CASI score (model 2), and between longitudinal changes in cholesterol levels and in CASI score (model 3). Goodness of fit among functions describing relationships between cholesterol levels and CASI score was assessed by calculating R-squared values. There is currently no definite threshold for R-squared values in the evaluation of models; however, it is a method to evaluate the significance of explanatory variables on the dependent variable [38]. We also compared the non-linear functions and linear functions in explaining the relationship between serum cholesterol and cognitive functions by comparing the generalized additive models introducing the restricted cubic spline terms (knots = 3) for serum cholesterol levels or not using splines function package of R. The significance of the regression models was evaluated using *F*-tests. We categorized longitudinal changes in TC, TG, HDL-c, and LDL-c into deciles and compared changes in CASI scores between individuals in first and tenth deciles (TC: 1st decile: over 35 mg/dL decrease, 10th decile: over 24 mg/dL increase; TG: 1st decile: over 47 mg/dL decrease, 10th decile: over 39.50 mg/dL increase; HDL-c: 1st decile: over 9 mg/dL decrease, 10th decile: over 10.00 mg/dL increase; LDL-c: 1st decile: over 30 mg/dL decrease, 10th decile: over 22.50 mg/dL increase) in order to assess effects of extreme increases and decreases in these parameters on cognitive function. Demographic and clinical characteristics were compared across subgroups (i.e., SMCI-S, SMCI-D, Dementia-S, Dementia-D) using one-way ANOVAs or chi-square tests. Multiple regression models were applied and compared across the subgroups. In sensitivity analysis, we repeated main analyses after excluding 357 individuals with SCD. The $\alpha$ levels were set as 0.016 according to Šidák correction for multiple comparisons. We did not correct the $\alpha$ levels for prediction using each variable in this study due to the explorative nature of the study [39, 40].

## Results

### Participants

Demographics and clinical characteristics of participants are presented in Table 1. Changes in cholesterol levels of participants are presented in Supplementary Table 1.

**Table 1:** Demographic and clinical characteristics among participant

	SMCI-S	SMCI-D	Dementia-S	Dementia-D	Statistical comparisons
*n*	857	396	454	427	
MCI (%)	64.03 (544/857)	88.89 (352/396)	--	--	${\chi }_{n=2134, df=1}^{2}$ = 90.96, *p* < 0.001
Age (yr)	63.48 (9.97)^*abc*^	76.03 (8.03)^*ae*^	76.27 (10.07)^*bf*^	78.86 (7.76)^*cef*^	*F*_*(3,2130)*_ = 115.47, *p* < 0.001
Follow-up duration (day)	1188.45 (542.45)^*abc*^	951.42 (408.21)^*ade*^	735.52 (304.17)^*bdf*^	484.74 (209.83)^*cef*^	*F*_*(3,2130)*_ = 296.76, *p* < 0.001
Educational level (yr)	6.45 (4.53)^*abc*^	4.80 (4.24)^*ae*^	4.55 (4.53)^*bf*^	3.74 (4.22)^*cef*^	*F*_*(3,2130)*_ = 42.75, *p* < 0.001
Sex (% male)	45.62 (391/857)^*c*^	46.97 (186/396)^*de*^	39.78 (181/454)^*d*^	38.88 (166/427)^*ce*^	${\chi }_{n=2134, df= 3}^{2}$ = 9.60, *p* < 0.05
Hypertension (%)	74.21 (636/857)^*a*^	83.33 (330/396)^*ade*^	74.45 (338/454)^*d*^	73.77 (315/427)^*e*^	${\chi }_{n=2134, df=3}^{2}$ = 14.86, *p* < 0.01
Diabetes mellitus (%)	32.32 (277/857)^*abc*^	41.92 (166/396)^*a*^	43.08 (196/454)^*b*^	42.86 (183/427)^*c*^	${\chi }_{n=2134, df=3}^{2}$ = 23.37, *p* < 0.001
Coronary artery disease (%)	11.09 (95/857)	14.39 (57/396)	8.79 (40/454)	9.60 (41/427)	${\chi }_{n=2134, df=3}^{2}$ = 7.78, *p* = 0.05
Cerebrovascular disease (%)	25.20 (216/857)	32.83 (130/396)	45.81 (208/454)	47.31 (202/427)	${\chi }_{n=2134, df=3}^{2}$ = 88.08, *p* < 0.001
Arrhythmia (%)	13.89 (119/857)	14.14 (56/396)	11.21 (51/454)	10.30 (44/427)	${\chi }_{n=2134, df=3}^{2}$ = 4.93, *p* = 0.15
Hypercholesterolemia (%)	49.59 (425/857)^*bc*^	49.75 (197/396)^*de*^	31.94(145/454)^*bd*^	33.72 (144/427)^*ce*^	${\chi }_{n=2134, df=3}^{2}$ = 60.23, *p* < 0.001
Anti-hypertensive (%)	57.64 (494/857)^*abc*^	64.65 (256/396)^*ade*^	34.58 (157/454)^*bd*^	40.52 (173/427)^*ce*^	${\chi }_{n=2134, df=3}^{2}$ = 112.24, *p* < 0.001
Anti-diabetic (%)	20.54 (176/857)^*a*^	29.80 (118/396)^*ad*^	21.10 (96/454)^*d*^	25.06 (107/427)	${\chi }_{n=2134, df=3}^{2}$ = 14.94, *p* < 0.01
Anti-platelets (%)	64.99 (557/857)^*abc*^	58.33 (231/396)^*a*^	56.39 (256/454)^*b*^	57.85 (247/427)^*c*^	${\chi }_{n=2134, df=3}^{2}$ = 12.49, *p* < 0.01
Anti-coagulants (%)	14.47 (124/857)	12.63 (50/396)	17.18 (78/454)	13.58 (58/427)	${\chi }_{n=2134, df=3}^{2}$ = 4.04, *p* = 0.26
Anti-lipid agents (%)	43.41 (372/857)^*bc*^	41.92 (166/396)^*de*^	26.21 (119/454)^*bd*^	26.70 (114/427)^*ce*^	${\chi }_{n=2134, df=3}^{2}$ = 61.24, *p* < 0.001
CASI (maximum score = 100)	76.73 (14.49)^*abc*^	66.23 (15.22)^*ade*^	41.85 (22.26)^*bd*^	39.94 (20.66)^*ce*^	*F*_*(3,2130)*_ = 603.99, *p* < 0.001
Change in CASI	-0.04 (6.67)^*abc*^	-11.72 (12.88)^*ad*^	2.21 (12.23)^*bdf*^	-10.99 (10.95)^*cf.*^	*F*_*(3,2130)*_ = 105.16, *p* < 0.001
HAI-ADL (maximum score = 43)	3.16 (2.54)^*abc*^	5.15 (2.96)^*ade*^	16.72 (6.79)^*bd*^	16.11 (6.70)^*ce*^	*F*_*(3,2130)*_ = 1212.17, *p* < 0.001
NPI-SB (maximum score = 144)	4.30 (6.09)^*bc*^	4.83 (6.29)^*de*^	12.12 (13.00)^*bd*^	11.36 (12.14)^*ce*^	*F*_*(3,2130)*_ = 105.16, *p* < 0.001
CCDR-SB (maximum score = 18)	1.36 (1.13)^*abc*^	2.36 (1.30)^*ade*^	9.11 (3.81)^*bd*^	8.82 (3.53)^*ce*^	*F*_*(3,2130)*_ = 105.16, *p* < 0.001
CFS (maximum = 7)	2.76 (2.25)^*b*^	2.58 (1.88)^*e*^	2.37 (1.50)^*bf*^	2.88 (2.25)^*ef*^	*F*_*(3,2130)*_ = 5.07, *p* < 0.001
TC (mg/dL)	175.82 (34.87)^*ac*^	165.40 (38.96)^*ad*^	173.99 (35.70)^*df*^	165.71 (38.47)^*cf.*^	*F*_*(3,2130)*_ = 115.47, *p* < 0.001
TG (mg/dL)	133.05 (88.98)^*a*^	118.32 (76.19)^*ad*^	136.49 (88.08)^*df*^	123.39 (74.77)^*f*^	*F*_*(3,2130)*_ = 4.51, *p* < 0.05
HDL-c (mg/dL)	53.50 (13.75)^*bc*^	51.86 (15.50)^*de*^	48.57 (13.15)^*bd*^	48.31 (13.74)^*ce*^	*F*_*(3,1567)*_ = 115.47, *p* < 0.001
LDL-c (mg/dL)	106.03 (30.04)^*ac*^	97.59 (33.05)^*ad*^	105.93 (30.59)^*df*^	99.98 (33.39)^*cf.*^	*F*_*(3,2126)*_ = 296.76, *p* < 0.001

CASI: Cognitive Assessment Screening Instrument; CDR-SB: Clinical Dementia Rating-Sum of boxes; CFS: Clinical Frailty Scale; Dementia-D: Individuals with progressive dementia; Dementia-S: Individuals with dementia who were not progressive; HAI-ADL: History-Based Artificial Intelligence-Activities of Daily Living; HDL-c: High-density lipoprotein cholesterol; LDL-c: Low-density lipoprotein cholesterol; MCI: Mild cognitive impairment; NPI-SB: Neuropsychiatric Inventory-Sum of boxes; SMCI-D: Subjective or mild cognitive impairment individuals who converted to dementia; SMCI-S: Stable subjective or mild cognitive impairment individuals; TC: Total cholesterol; TG: Triglyceride

^*a*^: SMCI-S $\ne$ SMCI-D; ^*b*^: SMCI-S $\ne$ Dementia-S; ^*c*^: SMCI-S $\ne$ Dementia-D; ^*d*^: SMCI-D $\ne$ Dementia-S; ^*e*^: SMCI-D $\ne$ Dementia-D; ^*f*^: Dementia-S $\ne$ Dementia-D

Numbers are denoted as mean (SD) or proportion (number)

### Association of serum cholesterol and cognitive function

**Fig. 2:**
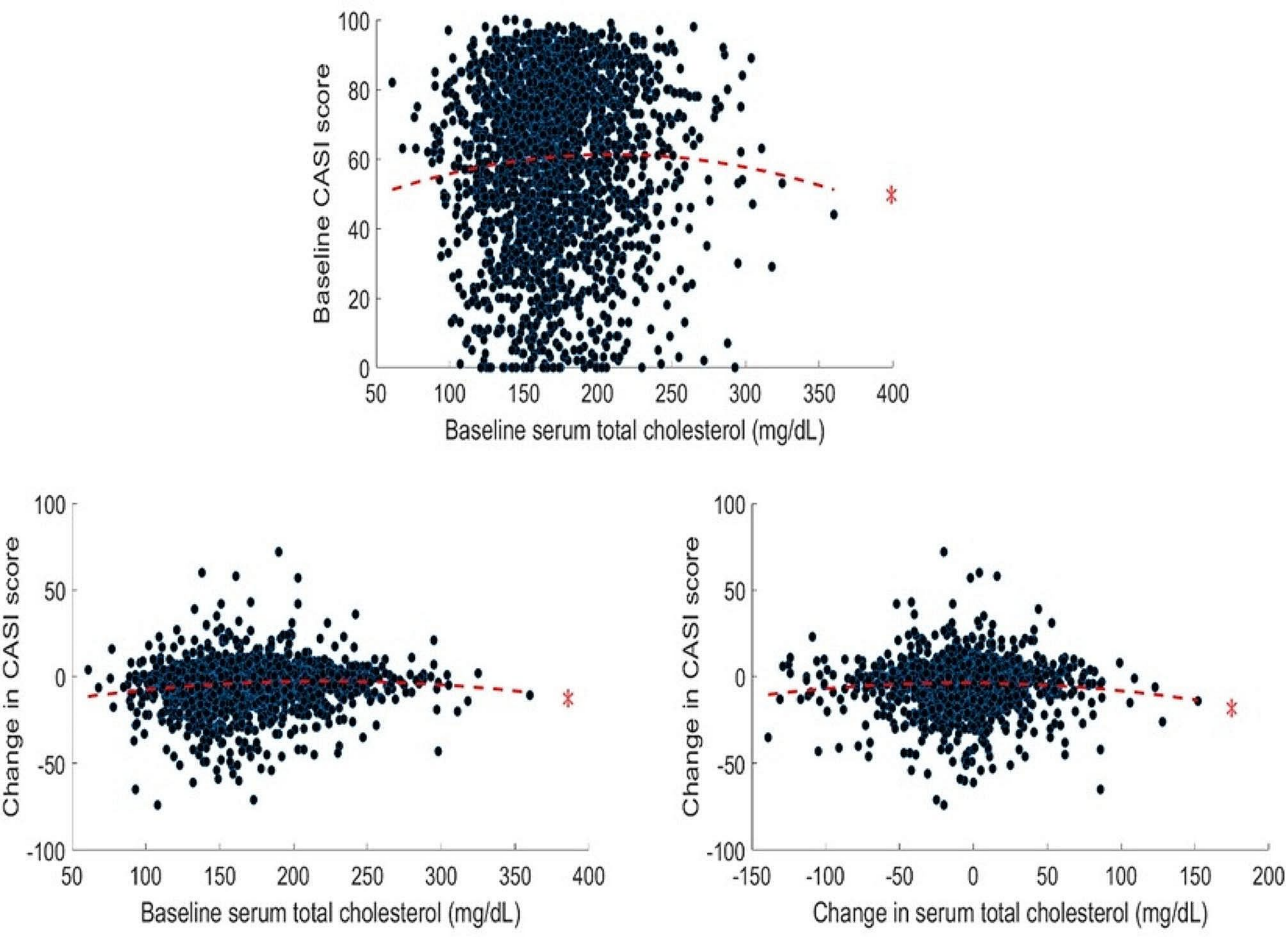
Relationship between serum TC levels and CASI scores. The upper panel denotes the relationship between baseline cholesterol levels and baseline CASI scores. The lower left plot denotes the relationship between baseline cholesterol levels and changes in CASI score. The lower right plot denotes the relationship between changes in cholesterol levels and CASI scores. Asterisk sign denotes a significant quadratic relationship. Abbreviations are the same as those used in Fig. 1

**Fig. 3:**
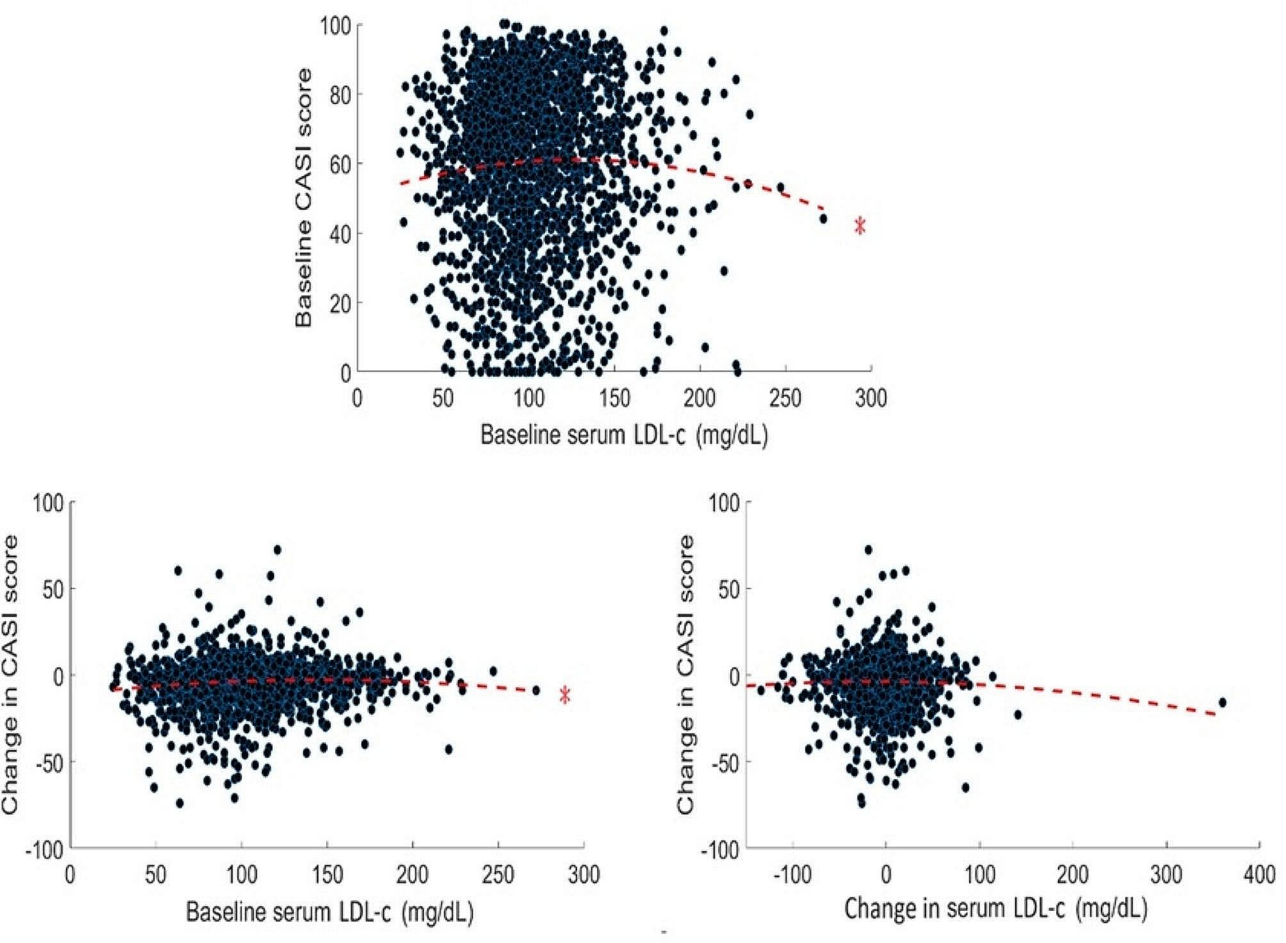
Relationship between serum LDL-c levels and CASI scores. LDL-c: Low-density lipoprotein cholesterol. Note and other abbreviations are the same as those used in Fig. 1

Baseline serum TC levels were linearly associated with maintenance or increase in CASI scores after controlling for covariates (*b* = 0.025, 95% confidence interval [CI] = 0.008–0.092, standard error [SE] = 0.01, *t* = 3.376, *p* < 0.01). Other linear relationships between serum cholesterol and CASI score were not significant (*p* = 0.162–0.994) (supplementary Table 2). Non-linear and quadratic inverted U-shaped functions could be used to describe the relationships between cholesterol parameters and cognitive function in all models (baseline: TC: baseline CASI, *R*^*2*^ = 0.003, | residuals | [1st quartile to 3rd quartile] = 10.12–29.01, *F* = 3.801, *p* < 0.016; change in CASI, *R*^*2*^ = 0.011, | residuals | = 11.90–30.24, *F* = 13.025, *p* < 0.001; HDL-c: baseline CASI, *R*^*2*^ = 0.008, | residuals | = 11.68–20.12, *F* = 6.342, *p* < 0.01; LDL-c: baseline CASI, *R*^*2*^ = 0.003, | residuals | = 13.14–20.26, *F* = 3.910, *p* < 0.050.016; change in CASI, *R*^*2*^ = 0.008, | residuals | = 11.23–17.10, *F* = 8.882, *p* < 0.001; longitudinal: TC: *R*^*2*^ = 0.003, | residuals | = 14.42–23.37, *F* = 3.923, *p* < 0.01 (Figs. 2 and 3), with the exceptions of relationship between HDL-c and changes in CASI (baseline: *R*^*2*^ = 0.005, | residuals | = 10.98–26.25, *F* = 3.331, *p* = 0.042; longitudinal: *R*^*2*^ = 0.006, | residuals | = 14.42–23.37, *F* = 4.460, *p* = 0.026) (supplementary Fig. 1), between TG and cognitive function (baseline: baseline CASI, *R*^*2*^ = 0.001, | residuals | = 10.32–30.11, *F* = 0.946, *p* = 0.381; change in CASI, *R*^*2*^ = 0.002, | residuals | = 10.63–21.49, *F* = 1.720, *p* = 0.198; longitudinal: *R*^*2*^ = 0.006, | residuals | = 8.26–19.19, *F* = 2.533, *p* = 0.102) (supplementary Fig. 2), and between changes in LDL-c and changes in CASI (*R*^*2*^ = 0.002, | residuals | = 15.32–22.38, *F* = 2.212, *p* = 0.146). The associations become insignificant after removing the cubic terms of serum cholesterol parameters in the models (*p* = 0.32–0.89), with the exceptions of the associations of baseline TC level and maintenance or increase of CASI scores and of baseline HDL-c and LDL-c and baseline CASI scores.

### Subgroup analyses

**Fig. 4:**
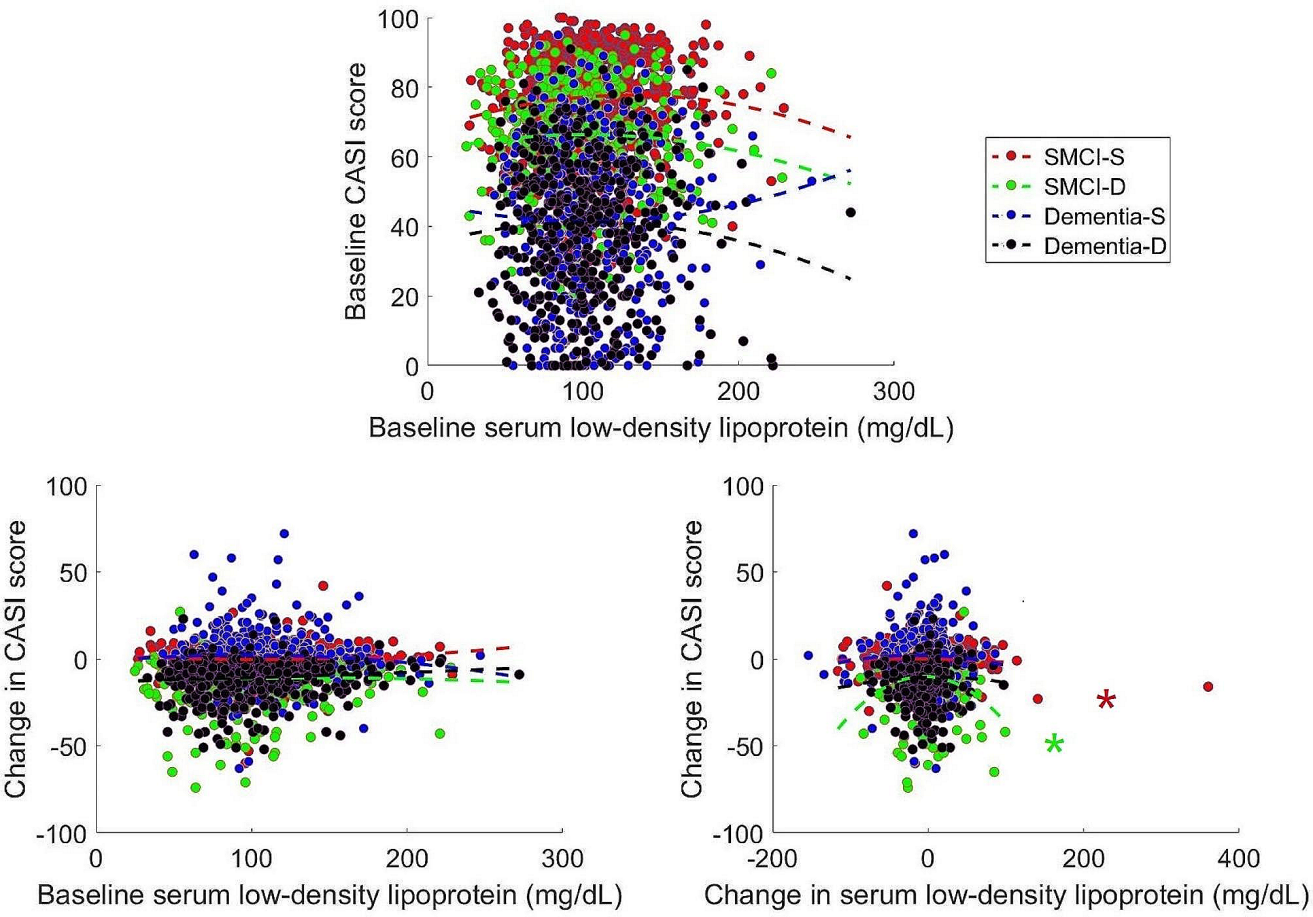
Relationship between serum LDL-c levels and CASI scores across individuals with different cognitive states. Note and abbreviations are the same as those used in Fig. 3

Serum cholesterol levels did not significantly nor linearly correlate with changes in CASI scores in each of the subgroups after controlling for covariates (TC: SMCI-S: *p* = 0.082–0.623; SMCI-D: *p* = 0.372–0.954; Dementia-S: *p* = 0.517–0.903; Dementia-D: *p* = 0.107 − 0.524; HDL-c: SMCI-S: *p* = 0.084–0.461; SMCI-D: *p* = 0.023–0.762; Dementia-S: *p* = 0.419–0.870; Dementia-D: *p* = 0.021–0.058; LDL-c: SMCI-S: *p* = 0.040–0.551; SMCI-D: *p* = 0.397–0.773; Dementia-S: *p* = 0.735–0.961; Dementia-D: *p* = 0.110–0.657; TG: SMCI-S: *p* = 0.036–0.130; SMCI-D: *p* = 0.062–0.314; Dementia-S: *p* = 0.822–0.995; Dementia-D: *p* = 0.084–0.862) (supplementary Tables 3–6). Relationships between changes in TC and changes in CASI scores (*R*^*2*^ = 0.064, | residuals | = 5.23–10.01, *F* = 13.706, *p* < 0.001) as well as between changes in TG and changes in CASI scores (*R*^*2*^ = 0.020, | residuals | = 9.71–17.20, *F* = 4.165, *p* < 0.016) could be explained by inverted U-shaped functions in the SMCI-D group (supplementary Figs. 3 and 4). Changes in LDL-c were also non-linearly and quadratically associated with changes in CASI scores with an inverted U-shaped relationship in SMCI-S (*R*^*2*^ = 0.059, | residuals | = 9.98–17.20, *F* = 12.447, *p* < 0.001) and SMCI-D (*R*^*2*^ = 0.059, | residuals | = 10.23–18.33, *F* = 12.447, *p* < 0.001) groups (Fig. 4). The relationship between changes in HDL-c and changes in CASI scores can also be explained by an inverted U-shaped function in Dementia-S group (*R*^*2*^ = 0.020, | residuals | = 10.10–18.23, *F* = 3.263, *p* < 0.016) (supplementary Fig. 5). The associations become insignificant after removing the cubic terms of serum cholesterol in the models (*p* = 0.21–0.78).

### Effects of extreme changes in cholesterol

There were no differences in level of changes in CASI scores between individuals in 1st decile and 10th decile groups of changes in cholesterol (TC, *p* = 0.266–0.73 L; HDL-c, *p* = 0.207–0.645; LDL-c, *p* = 0.355–0.972), with the exception of individuals with SMCI-S in 1st decile of TG changes displaying a slight but significant improvement in CASI scores over individuals with SMCI-S in 10th decile (1.438 vs. -1.154, *t*(202) = 2.275, *p* < 0.05) (total sample: *p* = 0.440; other subgroups: *p* = 0.420–0.823) (supplementary Fig. 6).

### Sensitivity analyses

Sensitivity analysis excluding individuals with SCD in the SMCI-S and SMCI-D groups revealed very similar results except for the results in terms of taking statins become insignificant (*p* = *0.302*) and males were associated with a higher baseline CASI scores (*p* < 0.016) in model 1 for LDL-c in the SMCI-D group. All other sensitivity analyses for fitting the quadratic functions remained the same for delineated relationships between cholesterol and cognitive function in the groups and individuals with extreme changes in cholesterol (*p* < 0.016).

## Discussion

In this study, we retrospectively investigated associations between serum cholesterol and cognitive function among individuals with different cognitive states. Baseline TC and LDL-c levels were linearly associated with maintenance or improvement in cognitive function after controlling for covariates. However, most associations between baseline or changes in cholesterol with cognitive function could be explained by non-linear, quadratic, and inverted U-shaped functions. The inverted U-shaped relationships were most evident between changes in TC, TG, and LDL-c in SMCI-D group and between HDL-c and cognitive function in Dementia-D group. No differences were observed in cognitive decline between individuals with drastic changes in serum cholesterol, with the exception of a mild improvement in cognitive function with an extreme TG decline in the SMCI-S group.

Guidelines for lipid control in CVD have mainly focused on lowering LDL-c [10, 11], however, associations between cholesterol and cognitive function have remained controversial [7, 12, 13, 41–43]. Previous research has reported deleterious effects of LDL-c on cognitive function [41, 44]. It appears that oxidized LDL-c promotes the development of atherosclerosis and chronic inflammation in the intima of arteries [45]. For example, one study reported that compared with LDL-c levels at 70.0–99.9 mg/dL, low LDL-c levels (< 70 mg/dL, especially < 55 mg/dL) were associated with significantly slower cognitive decline in a population-based setting [44]. There was, however, a non-linear trend for the effects of LDL-c on cognitive function in this study (i.e., a worse prognosis for cognitive function was observed among individuals with LDL-c levels between 70.0 and 99.9 mg/dL rather than higher or lower levels) [44].

Previous studies have focused on individuals with normal cognitive states and emphasized the importance of lowering LDL-c on health [46, 47]. Although this may be true among middle-aged individuals [48], our results indicate that decreases in LDL-c were only linearly associated with maintenance or improvement of cognition among older people with SMCI-S. Similarly, aggressively lowering TG appeared to cause a favorable effect on cognitive function for individuals with SMCI-S only, due perhaps to the fact that negative impacts of increases in LDL-c and TG on cognitive function are relatively more evident in early stages of cognitive decline. Furthermore, participants in this study were older than those in many previous studies. Protective effect of serum cholesterol on the preservation of cognitive decline might be more marked among older people than among younger people.

Consistent with other studies [1, 14, 15], we found that a non-linear and inverted U-shaped relationship could explain most of the relationships between serum cholesterol and cognitive function among older people, including the relationship between LDL-c and cognitive function. A recent study suggested that longitudinal increases in non-HDL-c may be protective for cognitive function among females or individuals without cardiovascular disease [43]. Similarly, a study reported that the risk for a decline in global cognitive (OR = 0.50) and memory function (OR = 0.45) was remarkably lower among older people with long-term increases in non-HDL-c than those in the consistently low level group [42]. Our results revealing an inverted U-shaped relationship between cholesterol and cognitive function might reflect the fact that cholesterol is important for myelination in the brain [49, 50]. Individuals with a low level or drastic decrease in cholesterol might suffer from reduced levels of myelination in the brain, especially in the early stages of cognitive decline.

Demyelination and axonal damage have recently been considered an important and potentially treatable neuropathology in AD [18, 51]. Previous studies have proposed complex interactive relationships between damage in myelin and amyloid in gray matter. Some studies have reported that demyelination occurs in the preclinical stages of AD, at a time when neurodegeneration is not apparent [25, 51, 52]. The findings of a more pronounced inverted U-shaped relationship between cholesterol and cognitive function in the SMCI-D group might reflect the fact that cholesterol plays a role in the process of demyelination in the early stages of AD. In addition, studies have found that sufficient levels of cholesterol may also prevent transmission or generation of AD pathologies [23, 25].

We found a non-linear and inverted-U relationship between changes in HDL-c and cognitive decline in Dementia-D group. Researchers have recently suggested that cholesterol also plays a role in preventing inflammation [45]. HDL-c may facilitate cholesterol efflux and inhibit the process of molecular adhesion during the formation of atherosclerotic plaques [45]. Previous studies have reported that cholesterol levels are correlated with cognitive decline during middle adulthood [48]. By contrast, cholesterol levels are negatively correlated with cognitive decline in late adulthood. The older age of the Dementia-D individuals in this study might be associated with the impacts of cholesterol changes in these individuals, especially changes in HDL-c and TG. The sensitivity of individuals in the Dementia-D group could be due to the fact that they are more prone to suffer from damage to blood vessels associated with atherosclerotic plaques [45]. In addition, the potentially poor brain state of these individuals may also contribute to sensitivity to HDL-c and TG changes [16].

This study shed new light on the relationship between cholesterol and cognitive function among older people. However, this study is subject to several limitations. First, the follow-up duration was relatively short; the full impact of changes in cholesterol levels on cognitive function could be better elucidated with longer follow-ups in future studies. Second, we did not collect information regarding treatment doses for CVD risks. Thus, the optimal level of cholesterol on cognitive function could be a result of proper pharmacological control of CVD risks [53, 54]. Third, the sample size in this study was relatively small whereby some of the subgroups may have been too small to detect any effects of cholesterol on cognitive function. Fourth, the present study could not completely exclude reverse causality. Individuals with cognitive decline could have exhibited changes in several factors that may have been associated with poor brain health (e.g., dietary habit, microbiota, or exercise habit) [55]. The possibility could cause the low explaining ability of the models in this study. Fifth, the prevalence of CVD risks among the excluded individuals was lower in this study. The relationship between serum cholesterol and cognitive functions may not be necessarily the same among individuals with better CVD risk management than current samples. Similarly, the current study included only the clinical samples. Baseline conditions, including the cognitive conditions, among the samples might be poorer than general population. Future studies could investigate these issues with a randomized control design and larger samples to further unravel the mechanisms of effects of cholesterol on brain and cognitive functions.

Most relationships between cholesterol and cognitive function conform to an inverted U-shaped function among older people. The inverted U-shaped relationship between cognitive function and cholesterol may be most evident in the early stages of cognitive decline. A balance between reducing CVD risks and preserving cognitive function should always be taken into consideration in clinical practice. It is merited to consider for effects of cholesterol reduction on cognitive decline among older people.

### Electronic supplementary material

Below is the link to the electronic supplementary material.

Supplementary Material 1

## Data Availability

Data available on request due to privacy/ethical restrictions. Requests to access these datasets should be directed to H-T C, changht@cycu.edu.tw.
