# Supplementary material for: Non-linear relationship between serum cholesterol levels and cognitive change among older people in the preclinical and prodromal stages of dementia: a retrospective longitudinal study in Taiwan
Source: BMC Geriatr. 2024 May 30;24:474. doi: 10.1186/s12877-024-05030-0 (PMC11138028; doi:10.1186/s12877-024-05030-0)
Supplement: Supplementary file 1 — Supplementary Material 1 [file 12877_2024_5030_MOESM1_ESM.docx]

**Supplementary information**

**Non-linear relationship between serum cholesterol levels and cognitive change among older people in the preclinical and prodromal stages of dementia**

**in Taiwan**

**Supplementary Table 1. Changes in Cholesterol across Groups****.**

|  | **SMCI-S** | **SMCI-D** | **Dementia-S** | **Dementia-D** | **Statistical comparisons** |
| --- | --- | --- | --- | --- | --- |
| TC | -4.28 (27.99) | -3.15 (26.19) | -5.70 (29.05) | -6.07 (27.03) | *F_(3,2130)_* = 1.27, *p* = 0.28 |
| TG | -4.81 (61.76) | -4.01 (49.21) | -1.37 (63.16) | -2.24 (53.35) | *F_(3,2130)_* = 0.38, *p* = 0.77 |
| HDL-c | 0.34 (9.33) | -0.63 (9.55) | 0.84 (8.86) | -0.60 (8.94) | *F_(3,1567)_* = 1.82, *p* = 0.14 |
| LDL-c | -2.30 (30.17) | -1.57 (23.91) | -4.37 (26.40) | -5.22 (21.17) | *F_(3,2126)_* = 1.90, *p* =0.13 |
| *Note.* Numbers are denoted as mean (SD) or proportion (number). HDL-c: High-density lipoprotein cholesterol; LDL-c: Low-density lipoprotein cholesterol; TC: Total cholesterol; TG: Triglyceride. | | | | | |

**Supplementary Table 2. Multiple Regression using Serum Cholesterol and Covariates on Cognitive Function among Total Sample**

|  | Model 1 | | | Model 2 | | | Model 3 | | |
| --- | --- | --- | --- | --- | --- | --- | --- | --- | --- |
|  | *B* | SE | *p*-value | *b* | SE | *p*-value | *b* | SE | *p*-value |
| ***TC*** |  |  |  |  |  |  |  |  |  |
| Age*^1,2,3^* | **-0.561** | **0.057** | **0.005** | **-0.111** | **0.032** | **0.004** | **-0.116** | **0.032** | **0.006** |
| Educational level*^1^* | **1.743** | **0.121** | **0.006** | 0.026 | 0.076 | 0.845 | 0.037 | 0.075 | 0.695 |
| Sex*^1^* | **1.942** | **0.994** | **0.007** | -0.319 | 0.566 | 0.633 | -0.698 | 0.562 | 0.209 |
| Hypertension*^1^* | -3.016 | 1.310 | 0.058 | -1.036 | 0.749 | 0.170 | -1.047 | 0.755 | 0.167 |
| Diabetes*^1^* | -2.041 | 1.341 | 0.030 | 0.808 | 0.775 | 0.282 | 0.469 | 0.769 | 0.547 |
| Coronary heart disease | 2.435 | 1.466 | 0.100 | 0.276 | 0.841 | 0.714 | 0.276 | 0.840 | 0.748 |
| Cerebrovascular disease | -0.771 | 0.618 | 0.215 | -0.609 | 0.615 | 0.316 | -0.772 | 0.613 | 0.210 |
| Arrhythmia*^2,3^* | 1.714 | 1.373 | 0.217 | 1.671 | 0.784 | 0.031 | 1.553 | 0.791 | 0.049 |
| Anti-hypertensive*^1^* | **8.187** | **1.201** | **0.010** | 0.295 | 0.694 | 0.694 | 0.238 | 0.698 | 0.739 |
| Anti-diabetic | -2.128 | 1.605 | 0.188 | -0.875 | 0.925 | 0.369 | -0.942 | 0.929 | 0.305 |
| Anti-lipid agent*^1^* | **3.158** | **1.033** | **0.010** | 0.064 | 0.595 | 0.922 | 0.337 | 0.591 | 0.577 |
| Anti-platelet | 0.926 | 0.923 | 0.323 | 0.451 | 0.526 | 0.395 | 0.437 | 0.529 | 0.422 |
| Anti-coagulant | 0.386 | 1.266 | 0.772 | 0.182 | 0.729 | 0.810 | 0.230 | 0.733 | 0.757 |
| Frailty | -0.081 | 0.214 | 0.687 | -0.211 | 0.128 | 0.075 | -0.224 | 0.123 | 0.064 |
| Follow-up duration | 0.024 | 0.218 | 0.896 | -0.009 | 0.522 | 0.930 | -0.526 | 0.375 | 0.319 |
| Total cholesterol*^2^* | 0.009 | 0.015 | 0.950 | **0.029** | **0.014** | **0.001** | 0.009 | 0.017 | 0.889 |

**Supplementary Table 2. (Continued)**

|  | Model 1 | | | Model 2 | | | Model 3 | | |
| --- | --- | --- | --- | --- | --- | --- | --- | --- | --- |
|  | *b* | SE | *p*-value | *b* | SE | *p*-value | *b* | SE | *p*-value |
| ***HDL-c*** |  |  |  |  |  |  |  |  |  |
| Age*^1^* | **-0.426** | **0.046** | **0.008** | -0.003 | 0.031 | 0.633 | 0.011 | 0.032 | 0.675 |
| Educational level*^1^* | **1.375** | **0.098** | **0.008** | 0.078 | 0.057 | 0.147 | 0.074 | 0.060 | 0.179 |
| Sex*^1^* | **2.370** | **0.793** | **0.010** | -0.549 | 0.458 | 0.230 | -0.349 | 0.448 | 0.421 |
| Hypertension | -0.667 | 0.920 | 0.470 | 0.996 | 0.532 | 0.065 | 1.036 | 0.534 | 0.061 |
| Diabetes*^2,3^* | 0.639 | 1.291 | 0.621 | **-2.661** | **0.757** | **0.008** | **-2.586** | **0.753** | **0.004** |
| Coronary heart disease*^1^* | 2.517 | 1.185 | 0.035 | 0.183 | 0.690 | 0.805 | 0.213 | 0.693 | 0.768 |
| Cerebrovascular disease*^1^* | **-4.767** | **0.890** | **0.003** | 0.748 | 0.527 | 0.160 | 0.808 | 0.529 | 0.128 |
| Arrhythmia | 0.735 | 1.169 | 0.537 | -0.078 | 0.685 | 0.909 | -0.062 | 0.686 | 0.918 |
| Anti-hypertensive*^1,2,3^* | 1.763 | 0.852 | 0.040 | **-1.893** | **0.493** | **0.002** | **-1.889** | **0.495** | **0.006** |
| Anti-diabetic*^2,3^* | -0.872 | 1.463 | 0.553 | **1.947** | **0.856** | **0.023** | **2.000** | **0.856** | **0.019** |
| Anti-lipid agent*^1^* | **-2.367** | **0.828** | **0.009** | 0.501 | 0.486 | 0.303 | 0.472 | 0.482 | 0.336 |
| Anti-platelet | -0.240 | 0.742 | 0.745 | -0.323 | 0.435 | 0.454 | -0.313 | 0.433 | 0.467 |
| Anti-coagulant | 0.242 | 1.054 | 0.832 | -0.003 | 0.609 | 1.001 | -0.044 | 0.610 | 0.940 |
| Frailty*^1^* | **-1.679** | **0.051** | **0.002** | -0.019 | 0.034 | 0.411 | -0.010 | 0.027 | 0.560 |
| Follow-up duration | 0.029 | 0.337 | 0.624 | -0.024 | 0.212 | 0.555 | 0.005 | 0.055 | 0.826 |
| High-density lipoprotein*^1^* | 0.052 | 0.032 | 0.050 | 0.039 | 0.032 | 0.090 | 0.041 | 0.037 | 0.249 |

**Supplementary Table 2. (Continued)**

|  | Model 1 | | | Model 2 | | | Model 3 | | |
| --- | --- | --- | --- | --- | --- | --- | --- | --- | --- |
|  | *b* | SE | *p*-value | *b* | SE | *p*-value | *b* | SE | *p*-value |
| ***LDL-c*** |  |  |  |  |  |  |  |  |  |
| Age*^1,2,3^* | **0.679** | **0.040** | **0.004** | **-0.074** | **0.027** | **0.001** | **-0.074** | **0.022** | **0.001** |
| Educational level*^1^* | **0.175** | **0.079** | **0.007** | 0.040 | 0.051 | 0.435 | 0.042 | 0.058 | 0.426 |
| Sex*^1^* | **0.429** | **0.611** | **0.006** | -0.229 | 0.418 | 0.578 | -0.319 | 0.415 | 0.436 |
| Hypertension | 0.060 | 0.777 | 0.668 | -0.467 | 0.536 | 0.367 | -0.494 | 0.531 | 0.344 |
| Diabetes | 0.005 | 0.853 | 0.955 | -0.277 | 0.583 | 0.623 | -0.405 | 0.580 | 0.472 |
| Coronary heart disease | 0.776 | 0.954 | 0.091 | 0.364 | 0.646 | 0.583 | 0.339 | 0.650 | 0.602 |
| Cerebrovascular disease*^1^* | **-3.918** | **0.723** | **0.003** | -0.452 | 0.475 | 0.335 | -0.506 | 0.481 | 0.286 |
| Arrhythmia | 0.920 | 0.918 | 0.804 | 0.921 | 0.626 | 0.142 | 0.866 | 0.629 | 0.167 |
| Anti-hypertensive*^1^* | 0.007 | 0.757 | 0.032 | -0.231 | 0.513 | 0.640 | -0.241 | 0.510 | 0.634 |
| Anti-diabetic | 0.026 | 1.048 | 0.536 | -0.110 | 0.710 | 0.875 | -0.148 | 0.715 | 0.832 |
| Anti-lipid agent*^1^* | **0.339** | **0.681** | **0.014** | -0.142 | 0.465 | 0.756 | -0.065 | 0.457 | 0.890 |
| Anti-platelet | 0.467 | 0.590 | 0.438 | 0.476 | 0.395 | 0.240 | 0.451 | 0.395 | 0.261 |
| Anti-coagulant | 0.944 | 0.862 | 0.205 | -1.121 | 0.586 | 0.057 | -1.139 | 0.584 | 0.054 |
| Frailty*^1^* | **0.550** | **0.042** | **0.008** | 0.046 | 0.025 | 0.098 | 0.041 | 0.030 | 0.109 |
| Follow-up duration | 0.028 | 0.342 | 0.624 | -0.032 | 0.221 | 0.558 | 0.001 | 0.056 | 0.832 |
| Low-density lipoprotein*^2^* | 0.247 | 0.017 | 0.503 | 0.017 | 0.011 | 0.020 | 0.008 | 0.014 | 0.909 |

**Supplementary Table 2. (Continued)**

|  | Model 1 | | | Model 2 | | | Model 3 | | |
| --- | --- | --- | --- | --- | --- | --- | --- | --- | --- |
|  | *b* | SE | *p*-value | *b* | SE | *p*-value | *b* | SE | *p*-value |
| ***TG*** |  |  |  |  |  |  |  |  |  |
| Age*^1^* | **-0.023** | **0.007** | **0.009** | -0.023 | 0.024 | 0.249 | -0.020 | 0.029 | 0.256 |
| Educational level*^1^* | **0.148** | **0.012** | **0.010** | -0.066 | 0.052 | 0.141 | -0.068 | 0.048 | 0.141 |
| Sex | -0.001 | 0.068 | 0.966 | 0.430 | 0.394 | 0.282 | 0.430 | 0.389 | 0.286 |
| Hypertension*^3^* | 0.070 | 0.087 | 0.456 | 0.997 | 0.496 | 0.050 | 0.993 | 0.494 | 0.051 |
| Diabetes*^2,3^* | 0.108 | 0.118 | 0.373 | **-2.464** | **0.687** | **0.002** | **-2.468** | **0.688** | **0.001** |
| Coronary heart disease*^1^* | 0.258 | 0.114 | 0.022 | -0.734 | 0.669 | 0.268 | -0.742 | 0.662 | 0.268 |
| Cerebrovascular disease*^1^* | **-0.341** | **0.081** | **0.003** | 0.218 | 0.490 | 0.675 | 0.218 | 0.489 | 0.668 |
| Arrhythmia | 0.142 | 0.113 | 0.211 | -0.271 | 0.652 | 0.685 | -0.268 | 0.656 | 0.678 |
| Anti-hypertensive*^3^* | 0.079 | 0.080 | 0.336 | -2.038 | 0.469 | 0.008 | **-2.032** | **0.478** | **0.010** |
| Anti-diabetic*^2,3^* | -0.288 | 0.132 | 0.035 | **2.008** | **0.806** | **0.013** | **2.000** | **0.802** | **0.016** |
| Anti-lipid agent | -0.110 | 0.082 | 0.126 | 0.004 | 0.468 | 1.002 | -0.007 | 0.464 | 0.982 |
| Anti-platelet | 0.007 | 0.074 | 0.951 | -0.036 | 0.411 | 0.929 | -0.037 | 0.411 | 0.926 |
| Anti-coagulant | -0.050 | 0.103 | 0.545 | 1.040 | 0.600 | 0.085 | 1.036 | 0.602 | 0.087 |
| Frailty*^1^* | **-0.139** | **0.013** | **0.004** | -0.007 | 0.025 | 0.636 | -0.003 | 0.031 | 0.635 |
| Follow-up duration | 0.029 | 0.333 | 0.628 | -0.028 | 0.219 | 0.554 | 0.009 | 0.054 | 0.826 |
| Triglyceride | 0.005 | 0.007 | 0.066 | 0.004 | 0.008 | 0.753 | 0.010 | 0.004 | 0.878 |

*Note.* Model 1 used baseline cholesterol variable and covariates as predictors of baseline CASI score. Model 2 used baseline cholesterol variable and covariates as predictors of changes in CASI score. Model 3 used changes in cholesterol variable and covariates as predictors of changes in CASI score. Units of age and educational levels are years. Male was coded as 1 and female as 0. Vascular risk factors and their treatments were binary coded. Bold text font indicates significant predictor. *^1^*: Significant in Model 1. *^2^*: Significant in Model 2. *^3^*: Significant in Model 3. *Abbreviation. b*: Unstandardized weight. HDL-c: High-density lipoprotein cholesterol. LDL-c: Low-density lipoprotein cholesterol. SE: Standard error. TC: Total cholesterol. TG: Triglyceride.

**Supplementary Table 3. Linear Regression using TC and Covariates on Cognitive Function across Subgroups**

|  | Model 1 | | | Model 2 | | | Model 3 | | |
| --- | --- | --- | --- | --- | --- | --- | --- | --- | --- |
|  | *b* | SE | *p*-value | *b* | SE | *p*-value | *b* | SE | *p*-value |
| ***SMCI-S*** |  |  |  |  |  |  |  |  |  |
| Age*^1^* | **-0.228** | **0.049** | **0.005** | -0.006 | 0.029 | 0.625 | -0.008 | 0.031 | 0.606 |
| Educational level*^1^* | **1.589** | **0.116** | **0.003** | -0.003 | 0.068 | 0.907 | -0.001 | 0.065 | 0.879 |
| Sex | 0.285 | 0.874 | 0.760 | 0.555 | 0.502 | 0.274 | 0.439 | 0.496 | 0.379 |
| Hypertension | -1.225 | 1.310 | 0.345 | -0.825 | 0.750 | 0.272 | -0.920 | 0.752 | 0.223 |
| Diabetes*^2,3^* | -0.677 | 1.322 | 0.611 | 1.754 | 0.762 | 0.027 | 1.752 | 0.757 | 0.023 |
| Coronary heart disease | 0.188 | 1.317 | 0.896 | 0.776 | 0.755 | 0.310 | 0.811 | 0.755 | 0.283 |
| Cerebrovascular disease*^1^* | **-4.955** | **1.101** | **0.000** | 0.613 | 0.637 | 0.339 | 0.565 | 0.639 | 0.387 |
| Arrhythmia | -0.771 | 1.192 | 0.519 | 0.183 | 0.685 | 0.791 | 0.146 | 0.688 | 0.848 |
| Anti-hypertensive*^2,3^* | 1.191 | 1.216 | 0.328 | 1.501 | 0.702 | 0.036 | 1.545 | 0.703 | 0.033 |
| Anti-diabetic*^2,3^* | -2.237 | 1.586 | 0.162 | -1.804 | 0.914 | 0.055 | -1.874 | 0.908 | 0.042 |
| Anti-lipid agent | 0.498 | 0.874 | 0.572 | -0.454 | 0.507 | 0.371 | -0.392 | 0.502 | 0.434 |
| Anti-platelet*^1^* | -1.773 | 0.849 | 0.046 | 0.002 | 0.490 | 1.008 | -0.006 | 0.493 | 0.993 |
| Anti-coagulant*^2,3^* | 0.832 | 1.145 | 0.475 | -1.512 | 0.658 | 0.021 | -1.464 | 0.652 | 0.024 |
| Frailty | 0.030 | 0.339 | 0.629 | -0.022 | 0.221 | 0.561 | 0.004 | 0.059 | 0.829 |
| Follow-up duration | 0.022 | 0.342 | 0.622 | -0.024 | 0.213 | 0.555 | 0.009 | 0.055 | 0.825 |
| Total cholesterol | -0.002 | 0.021 | 0.623 | 0.010 | 0.013 | 0.161 | -0.014 | 0.017 | 0.082 |

**Supplementary Table 3. (Continued)**

|  | Model 1 | | | Model 2 | | | Model 3 | | |
| --- | --- | --- | --- | --- | --- | --- | --- | --- | --- |
|  | *b* | SE | *p*-value | *b* | SE | *p*-value | *b* | SE | *p*-value |
| ***SMCI-D*** |  |  |  |  |  |  |  |  |  |
| Age*^1^* | **-0.462** | **0.085** | **0.002** | 0.072 | 0.087 | 0.452 | 0.060 | 0.093 | 0.495 |
| Educational level*^1^* | **1.431** | **0.172** | **0.003** | -0.163 | 0.181 | 0.341 | -0.171 | 0.179 | 0.321 |
| Sex | 2.466 | 1.432 | 0.094 | -1.247 | 1.449 | 0.390 | -1.225 | 1.440 | 0.391 |
| Hypertension | -0.017 | 2.213 | 1.000 | -3.477 | 2.250 | 0.128 | -3.407 | 2.252 | 0.137 |
| Diabetes | -3.826 | 2.039 | 0.063 | 0.657 | 2.070 | 0.756 | 0.566 | 2.055 | 0.794 |
| Coronary heart disease | 2.658 | 1.908 | 0.168 | -0.466 | 1.933 | 0.815 | -0.423 | 1.929 | 0.831 |
| Cerebrovascular disease | -0.731 | 1.633 | 0.656 | -2.411 | 1.654 | 0.145 | -2.330 | 1.660 | 0.161 |
| Arrhythmia | 1.900 | 1.918 | 0.330 | -0.099 | 1.943 | 0.959 | -0.276 | 1.937 | 0.892 |
| Anti-hypertensive | 1.462 | 1.809 | 0.420 | 2.549 | 1.838 | 0.176 | 2.371 | 1.831 | 0.204 |
| Anti-diabetic | 3.834 | 2.244 | 0.095 | 0.376 | 2.282 | 0.877 | 0.323 | 2.269 | 0.897 |
| Anti-lipid agent | -0.640 | 1.428 | 0.651 | 1.603 | 1.452 | 0.278 | 1.796 | 1.431 | 0.219 |
| Anti-platelet | 0.309 | 1.346 | 0.823 | -0.193 | 1.367 | 0.889 | -0.250 | 1.367 | 0.856 |
| Anti-coagulant | -0.847 | 1.951 | 0.667 | 1.069 | 1.982 | 0.592 | 1.165 | 1.984 | 0.562 |
| Frailty | 0.024 | 0.341 | 0.628 | -0.031 | 0.220 | 0.557 | 0.010 | 0.059 | 0.828 |
| Follow-up duration | 0.026 | 0.334 | 0.623 | -0.022 | 0.212 | 0.556 | 0.006 | 0.054 | 0.828 |
| Total cholesterol | 0.000 | 0.026 | 0.954 | 0.014 | 0.024 | 0.783 | 0.027 | 0.025 | 0.372 |

**Supplementary Table 3. (Continued)**

|  | Model 1 | | | Model 2 | | | Model 3 | | |
| --- | --- | --- | --- | --- | --- | --- | --- | --- | --- |
|  | *b* | SE | *p*-value | *b* | SE | *p*-value | *b* | SE | *p*-value |
| ***Dementia-S*** |  |  |  |  |  |  |  |  |  |
| Age | -0.011 | 0.111 | 0.871 | 0.002 | 0.062 | 0.949 | 0.000 | 0.065 | 0.929 |
| Educational level*^1^* | **1.553** | **0.262** | **0.009** | -0.032 | 0.155 | 0.839 | -0.030 | 0.150 | 0.829 |
| Sex | 1.730 | 2.392 | 0.482 | 0.945 | 1.386 | 0.503 | 0.999 | 1.339 | 0.467 |
| Hypertension | -0.259 | 2.615 | 0.921 | 0.212 | 1.512 | 0.901 | 0.290 | 1.520 | 0.859 |
| Diabetes | 2.877 | 2.645 | 0.280 | 0.115 | 1.528 | 0.948 | 0.064 | 1.522 | 0.973 |
| Coronary heart disease | 6.282 | 3.610 | 0.084 | 2.507 | 2.087 | 0.240 | 2.552 | 2.088 | 0.232 |
| Cerebrovascular disease*^1^* | **-7.764** | **2.190** | **0.001** | 0.325 | 1.288 | 0.808 | 2.602 | 0.214 | 0.226 |
| Arrhythmia *^2,3^* | 0.436 | 3.254 | 0.895 | 4.030 | 1.879 | 0.041 | 4.012 | 1.883 | 0.038 |
| Anti-hypertensive | 1.413 | 2.700 | 0.606 | -1.635 | 1.563 | 0.302 | -1.649 | 1.559 | 0.293 |
| Anti-diabetic | -3.473 | 3.493 | 0.323 | 0.586 | 2.029 | 0.777 | 0.608 | 2.019 | 0.767 |
| Anti-lipid agent | 2.461 | 2.698 | 0.366 | -2.256 | 1.561 | 0.155 | -2.243 | 1.554 | 0.149 |
| Anti-platelet | -0.086 | 2.031 | 0.971 | 1.561 | 1.170 | 0.188 | 1.554 | 1.175 | 0.196 |
| Anti-coagulant | 0.720 | 2.698 | 0.800 | 0.081 | 1.556 | 0.963 | 0.099 | 1.549 | 0.954 |
| Frailty | 0.024 | 0.337 | 0.627 | -0.027 | 0.212 | 0.554 | 0.009 | 0.054 | 0.832 |
| Follow-up duration | 0.027 | 0.337 | 0.625 | -0.028 | 0.212 | 0.559 | 0.003 | 0.060 | 0.827 |
| Total cholesterol | 0.013 | 0.035 | 0.888 | 0.006 | 0.026 | 0.903 | -0.004 | 0.021 | 0.517 |

**Supplementary Table 3. (Continued)**

|  | Model 1 | | | Model 2 | | | Model 3 | | |
| --- | --- | --- | --- | --- | --- | --- | --- | --- | --- |
|  | *b* | SE | *p*-value | *b* | SE | *p*-value | *b* | SE | *p*-value |
| ***Dementia-D*** |  |  |  |  |  |  |  |  |  |
| Age | 0.030 | 0.134 | 0.836 | 0.049 | 0.079 | 0.603 | 0.028 | 0.079 | 0.734 |
| Educational level*^1^* | **1.355** | **0.261** | **0.006** | -0.083 | 0.145 | 0.534 | -0.076 | 0.144 | 0.557 |
| Sex*^1^* | **6.239** | **2.161** | **0.004** | -1.219 | 1.212 | 0.317 | -1.492 | 1.192 | 0.218 |
| Hypertension | 3.266 | 2.575 | 0.207 | -1.738 | 1.438 | 0.235 | -1.624 | 1.453 | 0.261 |
| Diabetes | -0.038 | 2.774 | 0.993 | -0.861 | 1.547 | 0.580 | -1.377 | 1.534 | 0.376 |
| Coronary heart disease | -0.905 | 3.318 | 0.789 | 1.259 | 1.857 | 0.508 | 0.855 | 1.858 | 0.649 |
| Cerebrovascular disease | -2.324 | 2.142 | 0.279 | -0.241 | 1.203 | 0.843 | -0.643 | 1.188 | 0.591 |
| Arrhythmia | 3.067 | 3.202 | 0.342 | 2.108 | 1.785 | 0.243 | 1.667 | 1.786 | 0.356 |
| Anti-hypertensive | 0.562 | 2.499 | 0.828 | 0.773 | 1.400 | 0.592 | 0.749 | 1.399 | 0.593 |
| Anti-diabetic | 1.550 | 3.351 | 0.646 | 1.996 | 1.870 | 0.293 | 2.214 | 1.875 | 0.246 |
| Anti-lipid agent | 1.478 | 2.438 | 0.552 | 2.626 | 1.362 | 0.059 | 2.992 | 1.357 | 0.029 |
| Anti-platelet | 0.664 | 1.942 | 0.734 | -0.612 | 1.090 | 0.578 | -0.578 | 1.091 | 0.594 |
| Anti-coagulant | 3.658 | 2.776 | 0.197 | -1.095 | 1.560 | 0.478 | -0.897 | 1.549 | 0.568 |
| Frailty | -0.424 | 0.427 | 0.318 | -0.257 | 0.238 | 0.273 | -0.211 | 0.244 | 0.360 |
| Follow-up duration | 0.030 | 0.333 | 0.621 | -0.024 | 0.213 | 0.557 | 0.002 | 0.054 | 0.828 |
| Total cholesterol | 0.023 | 0.026 | 0.524 | 0.032 | 0.020 | 0.107 | 0.031 | 0.023 | 0.154 |

*Abbreviation.* SMCI-D: Individuals with subjective or mild cognitive impairment and progression to dementia at the follow-up. SMCI-S: Individuals with subjective or mild cognitive impairment and no progression to dementia at the follow-up. Dementia-S: Individuals with dementia and no deterioration in functions at the follow-up. Dementia-D: Individuals with dementia and deterioration in functions at the follow-up. Notes and other abbreviations are the same as those used in supplementary Table 2.

**Supplementary Table 4. Linear Regression Using HDL-C and Covariates on Cognitive Function across Subgroups**

|  | Model 1 | | | Model 2 | | | Model 3 | | |
| --- | --- | --- | --- | --- | --- | --- | --- | --- | --- |
|  | *b* | SE | *p*-value | *b* | SE | *p*-value | *b* | SE | *p*-value |
| ***SMCI-S*** |  |  |  |  |  |  |  |  |  |
| Age*^1^* | **-0.265** | **0.048** | **0.000** | -0.032 | 0.028 | 0.085 | -0.029 | 0.028 | 0.105 |
| Educational level*^1^* | **1.475** | **0.102** | **0.008** | 0.002 | 0.052 | 0.911 | 0.008 | 0.049 | 0.976 |
| Sex*^3^* | 1.391 | 0.855 | 0.111 | -0.698 | 0.402 | 0.082 | -0.782 | 0.388 | 0.047 |
| Hypertension | 0.939 | 1.003 | 0.353 | -0.324 | 0.473 | 0.490 | -0.381 | 0.477 | 0.414 |
| Diabetes | -1.230 | 1.523 | 0.417 | 0.435 | 0.716 | 0.548 | 0.326 | 0.708 | 0.663 |
| Coronary heart disease | 1.198 | 1.305 | 0.360 | 0.550 | 0.608 | 0.371 | 0.564 | 0.609 | 0.370 |
| Cerebrovascular disease*^1^* | **-3.673** | **1.121** | **0.007** | -0.911 | 0.524 | 0.083 | -0.970 | 0.527 | 0.070 |
| Arrhythmia | -0.988 | 1.284 | 0.451 | -0.067 | 0.609 | 0.907 | -0.123 | 0.603 | 0.842 |
| Anti-hypertensive | -1.153 | 0.935 | 0.217 | -0.215 | 0.437 | 0.616 | -0.156 | 0.439 | 0.724 |
| Anti-diabetic | 0.625 | 1.728 | 0.720 | -0.922 | 0.802 | 0.249 | -0.908 | 0.804 | 0.256 |
| Anti-lipid agent | -0.759 | 0.856 | 0.377 | 0.049 | 0.398 | 0.910 | 0.064 | 0.399 | 0.882 |
| Anti-platelet | 1.016 | 0.839 | 0.227 | 0.151 | 0.396 | 0.707 | 0.128 | 0.394 | 0.746 |
| Anti-coagulant | -0.086 | 1.187 | 0.941 | -0.501 | 0.559 | 0.358 | -0.517 | 0.553 | 0.350 |
| Frailty*^1^* | **-1.041** | **0.124** | **0.001** | 0.076 | 0.060 | 0.219 | 0.071 | 0.065 | 0.234 |
| Follow-up duration | 0.022 | 0.341 | 0.622 | -0.031 | 0.214 | 0.561 | 0.006 | 0.054 | 0.826 |
| High-density lipoprotein1 | 0.050 | 0.028 | 0.071 | 0.012 | 0.015 | 0.461 | -0.032 | 0.030 | 0.084 |

**Supplementary Table 4. (Continued)**

|  | Model 1 | | | Model 2 | | | Model 3 | | |
| --- | --- | --- | --- | --- | --- | --- | --- | --- | --- |
|  | b | SE | p-value | b | SE | p-value | b | SE | p-value |
| ***SMCI-D*** |  |  |  |  |  |  |  |  |  |
| Age*^1^* | **-0.613** | **0.116** | **0.004** | 0.110 | 0.094 | 0.212 | 0.113 | 0.086 | 0.211 |
| Educational level*^1^* | **1.412** | **0.211** | **0.005** | 0.167 | 0.169 | 0.333 | 0.155 | 0.167 | 0.375 |
| Sex | 1.718 | 1.820 | 0.355 | -0.891 | 1.414 | 0.530 | -0.395 | 1.386 | 0.776 |
| Hypertension*^1,2,3^* | **-6.070** | **2.404** | **0.017** | **6.364** | **1.879** | **0.005** | **6.348** | **1.887** | **0.007** |
| Diabetes*^2,3^* | 0.895 | 3.090 | 0.775 | **-8.102** | **2.408** | **0.004** | **-8.227** | **2.425** | **0.005** |
| Coronary heart disease | 1.749 | 2.382 | 0.473 | 2.376 | 1.858 | 0.203 | 2.524 | 1.865 | 0.180 |
| Cerebrovascular disease*^1^* | -4.653 | 2.064 | 0.026 | 2.900 | 1.606 | 0.081 | 3.066 | 1.608 | 0.060 |
| Arrhythmia | 2.003 | 2.521 | 0.434 | -1.003 | 1.959 | 0.609 | -0.991 | 1.965 | 0.616 |
| Anti-hypertensive*^1,2,3^* | **8.738** | **1.902** | **0.009** | **-6.061** | **1.484** | **0.004** | **-5.826** | **1.481** | **0.008** |
| Anti-diabetic*^2,3^* | 0.280 | 3.342 | 0.938 | 6.668 | 2.602 | 0.012 | **6.969** | **2.604** | **0.010** |
| Anti-lipid agent | -3.014 | 1.782 | 0.094 | 0.846 | 1.384 | 0.549 | 0.728 | 1.393 | 0.608 |
| Anti-platelet | -0.466 | 1.838 | 0.801 | -0.397 | 1.435 | 0.779 | -0.637 | 1.431 | 0.652 |
| Anti-coagulant | 0.571 | 2.271 | 0.811 | -0.152 | 1.774 | 0.932 | -0.206 | 1.772 | 0.909 |
| Frailty | -0.410 | 0.234 | 0.075 | -0.064 | 0.186 | 0.712 | -0.061 | 0.188 | 0.730 |
| Follow-up duration | 0.031 | 0.337 | 0.622 | -0.024 | 0.219 | 0.560 | 0.001 | 0.054 | 0.823 |
| High-density lipoprotein | -0.015 | 0.051 | 0.652 | -0.057 | 0.047 | 0.113 | 0.036 | 0.100 | 0.762 |

**Supplementary Table 4. (Continued)**

|  | Model 1 | | | Model 2 | | | Model 3 | | |
| --- | --- | --- | --- | --- | --- | --- | --- | --- | --- |
|  | *b* | SE | p-value | *b* | SE | p-value | *b* | SE | p-value |
| ***Dementia-S*** |  |  |  |  |  |  |  |  |  |
| Age | -0.080 | 0.091 | 0.367 | 0.041 | 0.049 | 0.450 | 0.049 | 0.055 | 0.426 |
| Educational level*^1^* | **1.351** | **0.221** | **0.009** | 0.119 | 0.121 | 0.323 | 0.125 | 0.116 | 0.283 |
| Sex | 2.501 | 1.928 | 0.199 | 0.712 | 1.048 | 0.499 | 0.599 | 1.008 | 0.559 |
| Hypertension | 0.470 | 2.029 | 0.825 | -0.114 | 1.098 | 0.921 | -0.087 | 1.098 | 0.944 |
| Diabetes | -0.332 | 2.774 | 0.906 | 2.445 | 1.502 | 0.113 | 2.420 | 1.494 | 0.112 |
| Coronary heart disease*^1^* | 6.084 | 3.046 | 0.051 | -1.509 | 1.644 | 0.364 | -1.478 | 1.645 | 0.369 |
| Cerebrovascular disease*^1^* | **-6.808** | **1.813** | **0.006** | 1.563 | 1.002 | 0.125 | 1.615 | 0.996 | 0.111 |
| Arrhythmia | -0.292 | 2.772 | 0.924 | -1.346 | 1.509 | 0.376 | -1.318 | 1.505 | 0.381 |
| Anti-hypertensive | -1.231 | 2.201 | 0.576 | 1.117 | 1.198 | 0.357 | 1.124 | 1.192 | 0.355 |
| Anti-diabetic | 0.848 | 3.299 | 0.808 | -2.758 | 1.788 | 0.127 | -2.744 | 1.784 | 0.124 |
| Anti-lipid agent | -3.722 | 2.257 | 0.102 | 1.161 | 1.224 | 0.352 | 1.200 | 1.228 | 0.332 |
| Anti-platelet | 0.882 | 1.696 | 0.606 | 0.228 | 0.919 | 0.812 | 0.278 | 0.922 | 0.770 |
| Anti-coagulant | 2.397 | 2.528 | 0.347 | -2.063 | 1.374 | 0.138 | -2.020 | 1.361 | 0.137 |
| Frailty*^1^* | **-1.426** | **0.120** | **0.003** | -0.016 | 0.067 | 0.785 | -0.015 | 0.067 | 0.777 |
| Follow-up duration | 0.023 | 0.338 | 0.622 | -0.029 | 0.217 | 0.553 | 0.000 | 0.061 | 0.830 |
| High-density lipoprotein | -0.023 | 0.062 | 0.630 | 0.011 | 0.036 | 0.870 | 0.069 | 0.081 | 0.419 |

**Supplementary Table 4. (Continued)**

|  | Model 1 | | | Model 2 | | | Model 3 | | |
| --- | --- | --- | --- | --- | --- | --- | --- | --- | --- |
|  | *b* | SE | p-value | *b* | SE | p-value | *b* | SE | p-value |
| ***Dementia-D*** |  |  |  |  |  |  |  |  |  |
| Age | -0.181 | 0.124 | 0.128 | 0.007 | 0.084 | 0.988 | 0.013 | 0.090 | 0.940 |
| Educational level*^1^* | **0.842** | **0.235** | **0.007** | 0.131 | 0.156 | 0.418 | 0.110 | 0.159 | 0.486 |
| Sex*^1^* | **6.023** | **1.951** | **0.006** | -0.400 | 1.323 | 0.763 | 0.167 | 1.293 | 0.904 |
| Hypertension | -0.363 | 2.167 | 0.873 | 0.778 | 1.466 | 0.602 | 1.095 | 1.470 | 0.460 |
| Diabetes*^1,2,3^* | 7.161 | 2.970 | 0.019 | **-8.490** | **2.003** | **0.008** | **-8.283** | **2.005** | **0.005** |
| Coronary heart disease | 4.402 | 3.076 | 0.162 | -1.251 | 2.074 | 0.545 | -1.073 | 2.084 | 0.612 |
| Cerebrovascular disease | 0.139 | 2.039 | 0.956 | 0.460 | 1.373 | 0.746 | 0.671 | 1.374 | 0.635 |
| Arrhythmia | 1.409 | 3.135 | 0.659 | 2.337 | 2.114 | 0.274 | 2.410 | 2.116 | 0.257 |
| Anti-hypertensive*^1,2,3^* | 5.176 | 2.163 | 0.022 | **-4.947** | **1.458** | **0.002** | **-5.136** | **1.470** | **0.005** |
| Anti-diabetic*^2,3^* | -3.490 | 3.487 | 0.324 | **8.083** | **2.351** | **0.004** | **8.217** | **2.358** | **0.006** |
| Anti-lipid agent*^1^* | **-6.172** | **2.270** | **0.013** | 0.332 | 1.535 | 0.837 | 0.395 | 1.529 | 0.810 |
| Anti-platelet | -3.318 | 1.790 | 0.071 | -1.719 | 1.216 | 0.164 | -1.698 | 1.216 | 0.161 |
| Anti-coagulant | -1.852 | 2.601 | 0.482 | 2.323 | 1.750 | 0.191 | 2.125 | 1.758 | 0.230 |
| Frailty*^1^* | **-1.205** | **0.127** | **0.002** | 0.046 | 0.091 | 0.662 | 0.062 | 0.086 | 0.526 |
| Follow-up duration | 0.023 | 0.336 | 0.628 | -0.028 | 0.218 | 0.553 | 0.005 | 0.054 | 0.825 |
| High-density lipoprotein*^1,2,^* | 0.153 | 0.067 | 0.021 | -0.084 | 0.044 | 0.028 | 0.142 | 0.080 | 0.058 |

Notes and abbreviations are the same as those used in supplementary Tables 2 and 3.

**Supplementary Table 5. Linear Regression Using LDL-C and Covariates on Cognitive Function across Subgroups**

|  | Model 1 | | | Model 2 | | | Model 3 | | |
| --- | --- | --- | --- | --- | --- | --- | --- | --- | --- |
|  | *b* | SE | *p*-value | *b* | SE | *p*-value | *b* | SE | *p*-value |
| ***SMCI-S*** |  |  |  |  |  |  |  |  |  |
| Age*^1^* | **-0.259** | **0.046** | **0.007** | -0.019 | 0.022 | 0.284 | -0.017 | 0.019 | 0.261 |
| Educational level*^1^* | **1.492** | **0.091** | **0.008** | 0.026 | 0.042 | 0.712 | 0.024 | 0.049 | 0.722 |
| Sex | 1.140 | 0.742 | 0.130 | 0.313 | 0.355 | 0.395 | 0.318 | 0.357 | 0.386 |
| Hypertension | -0.663 | 1.047 | 0.522 | -0.301 | 0.512 | 0.543 | -0.369 | 0.514 | 0.461 |
| Diabetes | -0.745 | 1.126 | 0.511 | 0.959 | 0.548 | 0.083 | 0.974 | 0.543 | 0.077 |
| Coronary heart disease | 0.201 | 1.153 | 0.869 | 1.020 | 0.564 | 0.077 | 1.039 | 0.566 | 0.068 |
| Cerebrovascular disease*^1^* | **-5.292** | **1.012** | **0.008** | 0.150 | 0.496 | 0.770 | 0.170 | 0.494 | 0.741 |
| Arrhythmia | -0.695 | 1.120 | 0.533 | 0.585 | 0.538 | 0.289 | 0.514 | 0.548 | 0.353 |
| Anti-hypertensive | 0.721 | 0.980 | 0.471 | 0.576 | 0.480 | 0.227 | 0.589 | 0.478 | 0.217 |
| Anti-diabetic | -1.712 | 1.371 | 0.217 | -0.664 | 0.661 | 0.314 | -0.666 | 0.664 | 0.315 |
| Anti-lipid agent | -0.693 | 0.767 | 0.370 | -0.298 | 0.378 | 0.417 | -0.280 | 0.370 | 0.444 |
| Anti-platelet | 0.310 | 0.729 | 0.675 | 0.583 | 0.351 | 0.108 | 0.548 | 0.349 | 0.120 |
| Anti-coagulant | -0.008 | 1.074 | 0.994 | -0.940 | 0.523 | 0.070 | -0.869 | 0.522 | 0.096 |
| Frailty*^1,2,3^* | **-1.254** | **0.117** | **0.005** | **0.213** | **0.054** | **0.009** | **0.212** | **0.057** | **0.007** |
| Follow-up duration | 0.024 | 0.333 | 0.621 | -0.026 | 0.220 | 0.561 | 0.010 | 0.060 | 0.832 |
| Low-density lipoprotein*^3^* | -0.006 | 0.014 | 0.551 | 0.011 | 0.010 | 0.336 | -0.012 | 0.009 | 0.040 |

**Supplementary Table 5. (Continued)**

|  | Model 1 | | | Model 2 | | | Model 3 | | |
| --- | --- | --- | --- | --- | --- | --- | --- | --- | --- |
|  | *b* | SE | *p*-value | *b* | SE | *p*-value | *b* | SE | *p*-value |
| ***SMCI-D*** |  |  |  |  |  |  |  |  |  |
| Age*^1^* | **-0.410** | **0.075** | **0.007** | 0.138 | 0.078 | 0.063 | 0.133 | 0.074 | 0.064 |
| Educational level*^1^* | **1.601** | **0.151** | **0.008** | -0.049 | 0.143 | 0.731 | -0.047 | 0.138 | 0.718 |
| Sex | 1.446 | 1.252 | 0.251 | -1.503 | 1.166 | 0.206 | -1.534 | 1.173 | 0.195 |
| Hypertension*^2,3^* | 1.025 | 1.825 | 0.577 | -3.481 | 1.696 | 0.049 | -3.524 | 1.700 | 0.041 |
| Diabetes | -1.845 | 1.863 | 0.326 | -0.368 | 1.733 | 0.828 | -0.487 | 1.726 | 0.785 |
| Coronary heart disease | 2.081 | 1.697 | 0.219 | 0.231 | 1.580 | 0.895 | 0.191 | 1.577 | 0.908 |
| Cerebrovascular disease | -0.594 | 1.508 | 0.694 | -2.517 | 1.396 | 0.075 | -2.602 | 1.401 | 0.071 |
| Arrhythmia | 0.489 | 1.787 | 0.789 | -1.813 | 1.663 | 0.274 | -1.825 | 1.667 | 0.280 |
| Anti-hypertensive | 1.532 | 1.570 | 0.332 | 2.071 | 1.455 | 0.164 | 2.009 | 1.452 | 0.175 |
| Anti-diabetic | 2.897 | 2.087 | 0.170 | 0.280 | 1.945 | 0.887 | 0.192 | 1.942 | 0.923 |
| Anti-lipid agent*^1^* | -2.967 | 1.260 | 0.020 | 0.873 | 1.177 | 0.463 | 0.963 | 1.166 | 0.418 |
| Anti-platelet | 1.405 | 1.253 | 0.263 | -0.105 | 1.166 | 0.931 | -0.151 | 1.169 | 0.897 |
| Anti-coagulant | 2.149 | 1.657 | 0.203 | -2.396 | 1.551 | 0.122 | -2.403 | 1.552 | 0.125 |
| Frailty*^1^* | **-0.623** | **0.166** | **0.005** | 0.012 | 0.155 | 0.948 | 0.007 | 0.152 | 1.003 |
| Follow-up duration | 0.026 | 0.338 | 0.629 | -0.029 | 0.217 | 0.561 | 0.004 | 0.061 | 0.831 |
| Low-density lipoprotein | 0.014 | 0.018 | 0.773 | 0.022 | 0.023 | 0.397 | -0.010 | 0.028 | 0.606 |

**Supplementary Table 5. (Continued)**

|  | Model 1 | | | Model 2 | | | Model 3 | | |
| --- | --- | --- | --- | --- | --- | --- | --- | --- | --- |
|  | *b* | SE | *p*-value | *b* | SE | *p*-value | *b* | SE | *p*-value |
| ***Dementia-S*** |  |  |  |  |  |  |  |  |  |
| Age | -0.029 | 0.082 | 0.657 | -0.020 | 0.050 | 0.629 | -0.015 | 0.051 | 0.624 |
| Educational level*^1^* | **1.511** | **0.182** | **0.007** | -0.061 | 0.112 | 0.528 | -0.070 | 0.121 | 0.521 |
| Sex | 0.794 | 1.554 | 0.619 | 1.028 | 0.964 | 0.298 | 1.040 | 0.960 | 0.289 |
| Hypertension | 0.614 | 1.737 | 0.732 | 0.680 | 1.077 | 0.540 | 0.684 | 1.084 | 0.535 |
| Diabetes | 0.064 | 1.898 | 0.976 | 0.495 | 1.177 | 0.687 | 0.510 | 1.166 | 0.668 |
| Coronary heart disease | 4.278 | 2.641 | 0.113 | 1.680 | 1.642 | 0.309 | 1.695 | 1.634 | 0.303 |
| Cerebrovascular disease*^1^* | **-5.299** | **1.587** | **0.004** | 0.843 | 0.991 | 0.403 | 0.847 | 0.987 | 0.406 |
| Arrhythmia | -0.347 | 2.278 | 0.880 | 2.411 | 1.415 | 0.094 | 2.420 | 1.410 | 0.097 |
| Anti-hypertensive | 0.744 | 1.892 | 0.700 | -2.174 | 1.179 | 0.064 | -2.182 | 1.167 | 0.063 |
| Anti-diabetic | 0.224 | 2.602 | 0.935 | 0.964 | 1.617 | 0.557 | 0.967 | 1.613 | 0.559 |
| Anti-lipid agent | -3.488 | 2.006 | 0.083 | -1.335 | 1.244 | 0.284 | -1.333 | 1.239 | 0.282 |
| Anti-platelet | 0.045 | 1.422 | 0.979 | 1.205 | 0.884 | 0.174 | 1.195 | 0.889 | 0.184 |
| Anti-coagulant | 0.625 | 2.233 | 0.783 | 0.256 | 1.386 | 0.859 | 0.252 | 1.392 | 0.865 |
| Frailty*^1^* | **-1.550** | **0.100** | **0.006** | 0.085 | 0.059 | 0.151 | 0.093 | 0.064 | 0.154 |
| Follow-up duration | 0.029 | 0.342 | 0.630 | -0.028 | 0.221 | 0.560 | 0.001 | 0.058 | 0.829 |
| Low-density lipoprotein | -0.001 | 0.028 | 0.822 | 0.000 | 0.023 | 0.924 | 0.000 | 0.028 | 0.995 |

**Supplementary Table 5. (Continued)**

|  | Model 1 | | | Model 2 | | | Model 3 | | |
| --- | --- | --- | --- | --- | --- | --- | --- | --- | --- |
|  | *b* | SE | *p*-value | *b* | SE | *p*-value | *b* | SE | *p*-value |
| ***Dementia-D*** |  |  |  |  |  |  |  |  |  |
| Age | -0.065 | 0.110 | 0.454 | 0.083 | 0.063 | 0.217 | 0.067 | 0.064 | 0.283 |
| Educational level*^1^* | **1.097** | **0.199** | **0.006** | -0.012 | 0.121 | 0.894 | -0.010 | 0.116 | 0.889 |
| Sex*^1^* | **6.433** | **1.661** | **0.008** | -0.738 | 0.988 | 0.459 | -0.772 | 0.979 | 0.435 |
| Hypertension | 1.693 | 1.906 | 0.375 | -0.732 | 1.135 | 0.523 | -0.640 | 1.139 | 0.576 |
| Diabetes*^2,3^* | 3.341 | 2.171 | 0.132 | **-3.278** | **1.297** | **0.019** | -3.380 | 1.291 | 0.017 |
| Coronary heart disease | 1.733 | 2.715 | 0.524 | 0.036 | 1.620 | 0.996 | -0.313 | 1.616 | 0.852 |
| Cerebrovascular disease | -1.110 | 1.748 | 0.530 | -0.795 | 1.043 | 0.442 | -0.881 | 1.030 | 0.389 |
| Arrhythmia | 1.041 | 2.719 | 0.708 | 1.007 | 1.625 | 0.542 | 0.861 | 1.610 | 0.598 |
| Anti-hypertensive | 2.063 | 1.981 | 0.307 | -0.535 | 1.188 | 0.652 | -0.533 | 1.176 | 0.654 |
| Anti-diabetic | 0.368 | 2.767 | 0.904 | 3.056 | 1.652 | 0.071 | 3.208 | 1.650 | 0.061 |
| Anti-lipid agent | -2.881 | 2.090 | 0.175 | 0.603 | 1.245 | 0.634 | 0.787 | 1.240 | 0.537 |
| Anti-platelet*^1^* | **-3.700** | **1.530** | **0.020** | 0.477 | 0.914 | 0.608 | 0.472 | 0.908 | 0.611 |
| Anti-coagulant | 2.474 | 2.357 | 0.302 | -1.658 | 1.403 | 0.241 | -1.652 | 1.407 | 0.242 |
| Frailty*^1^* | **-1.315** | **0.110** | **0.002** | 0.069 | 0.073 | 0.307 | 0.073 | 0.071 | 0.265 |
| Follow-up duration | 0.023 | 0.338 | 0.631 | -0.027 | 0.212 | 0.556 | 0.009 | 0.060 | 0.832 |
| Low-density lipoprotein | 0.001 | 0.024 | 0.862 | 0.015 | 0.023 | 0.665 | 0.053 | 0.027 | 0.084 |

Notes and abbreviations are the same as those used in supplementary Tables 2 and 3.

**Supplementary Table 6. Linear Regression Using TG and Covariates on Cognitive Function in across Subgroups**

|  | Model 1 | | | Model 2 | | | Model 3 | | |
| --- | --- | --- | --- | --- | --- | --- | --- | --- | --- |
|  | *b* | SE | *p*-value | *b* | SE | *p*-value | *b* | SE | *p*-value |
| ***SMCI-S*** |  |  |  |  |  |  |  |  |  |
| Age*^1^* | **-0.019** | **0.012** | **0.000** | -0.014 | 0.021 | 0.416 | -0.011 | 0.026 | 0.485 |
| Educational level*^1^* | **0.147** | **0.017** | **0.003** | -0.028 | 0.041 | 0.443 | -0.022 | 0.050 | 0.437 |
| Sex | 0.033 | 0.079 | 0.719 | 0.065 | 0.338 | 0.854 | 0.057 | 0.341 | 0.879 |
| Hypertension | -0.022 | 0.104 | 0.799 | 0.237 | 0.441 | 0.607 | 0.212 | 0.442 | 0.640 |
| Diabetes*^3^* | -0.144 | 0.154 | 0.316 | 0.195 | 0.658 | 0.774 | 0.185 | 0.659 | 0.781 |
| Coronary heart disease | 0.186 | 0.132 | 0.157 | -0.404 | 0.581 | 0.476 | -0.426 | 0.570 | 0.453 |
| Cerebrovascular disease*^1^* | **-0.389** | **0.117** | **0.003** | 0.658 | 0.497 | 0.192 | 0.633 | 0.501 | 0.208 |
| Arrhythmia | 0.024 | 0.129 | 0.904 | 0.432 | 0.569 | 0.445 | 0.438 | 0.566 | 0.441 |
| Anti-hypertensive | -0.092 | 0.089 | 0.286 | 0.217 | 0.407 | 0.598 | 0.229 | 0.408 | 0.570 |
| Anti-diabetic | 0.043 | 0.167 | 0.815 | -0.696 | 0.756 | 0.357 | -0.699 | 0.751 | 0.351 |
| Anti-lipid agent | -0.147 | 0.088 | 0.070 | 0.235 | 0.380 | 0.555 | 0.194 | 0.376 | 0.608 |
| Anti-platelet*^3^* | 0.139 | 0.080 | 0.090 | -0.689 | 0.360 | 0.058 | -0.705 | 0.362 | 0.048 |
| Anti-coagulant | -0.019 | 0.122 | 0.869 | 0.112 | 0.530 | 0.847 | 0.110 | 0.534 | 0.855 |
| Frailty*^1^* | **-0.079** | **0.020** | **0.003** | -0.032 | 0.060 | 0.549 | -0.030 | 0.059 | 0.509 |
| Follow-up duration | 0.031 | 0.340 | 0.624 | -0.025 | 0.220 | 0.562 | 0.001 | 0.054 | 0.831 |
| Triglyceride*^1,2,3^* | 0.006 | 0.010 | 0.597 | 0.000 | 0.011 | 0.811 | -0.001 | 0.008 | 0.501 |

**Supplementary Table 6. (Continued)**

|  | Model 1 | | | Model 2 | | | Model 3 | | |
| --- | --- | --- | --- | --- | --- | --- | --- | --- | --- |
|  | *b* | SE | *p*-value | *b* | SE | *p*-value | *b* | SE | *p*-value |
| ***SMCI-D*** |  |  |  |  |  |  |  |  |  |
| Age*^1^* | **-0.024** | **0.017** | **0.012** | 0.058 | 0.082 | 0.504 | 0.055 | 0.087 | 0.521 |
| Educational level*^1,2,3^* | **0.187** | **0.022** | **0.005** | -0.338 | 0.143 | 0.027 | -0.339 | 0.147 | 0.018 |
| Sex | -0.007 | 0.153 | 0.940 | 1.478 | 1.205 | 0.219 | 1.617 | 1.204 | 0.188 |
| Hypertension | -0.065 | 0.206 | 0.733 | 0.021 | 1.689 | 1.001 | -0.030 | 1.689 | 0.988 |
| Diabetes*^2,3^* | 0.377 | 0.255 | 0.146 | -5.072 | 2.103 | 0.024 | -5.104 | 2.093 | 0.023 |
| Coronary heart disease | 0.204 | 0.214 | 0.360 | -1.783 | 1.769 | 0.313 | -1.835 | 1.763 | 0.303 |
| Cerebrovascular disease | -0.177 | 0.186 | 0.321 | -1.997 | 1.527 | 0.192 | 0.635 | 0.500 | 0.207 |
| Arrhythmia | 0.109 | 0.230 | 0.643 | -2.459 | 1.877 | 0.193 | -2.359 | 1.873 | 0.214 |
| Anti-hypertensive*^1,2,3^* | **0.790** | **0.171** | **0.001** | **-6.269** | **1.368** | **0.008** | **-6.509** | **1.370** | **0.009** |
| Anti-diabetic*^1,2,3^* | **-0.729** | **0.289** | **0.013** | **7.143** | **2.333** | **0.006** | **6.987** | **2.323** | **0.009** |
| Anti-lipid agent | -0.275 | 0.163 | 0.083 | 0.767 | 1.316 | 0.571 | 0.802 | 1.307 | 0.553 |
| Anti-platelet | 0.060 | 0.160 | 0.712 | 1.999 | 1.280 | 0.121 | 2.116 | 1.280 | 0.108 |
| Anti-coagulant | -0.053 | 0.210 | 0.794 | 1.733 | 1.676 | 0.310 | 1.793 | 1.668 | 0.286 |
| Frailty | -0.020 | 0.023 | 0.237 | -0.233 | 0.171 | 0.154 | -0.234 | 0.173 | 0.153 |
| Follow-up duration | 0.027 | 0.338 | 0.628 | -0.029 | 0.212 | 0.555 | 0.005 | 0.057 | 0.830 |
| Triglyceride*^1,3^* | 0.002 | 0.002 | 0.042 | -0.002 | 0.006 | 0.059 | 0.026 | 0.013 | 0.025 |

**Supplementary Table 6. (Continued)**

|  | Model 1 | | | Model 2 | | | Model 3 | | |
| --- | --- | --- | --- | --- | --- | --- | --- | --- | --- |
|  | *b* | SE | *p*-value | *b* | SE | *p*-value | *b* | SE | *p*-value |
| ***Dementia-S*** |  |  |  |  |  |  |  |  |  |
| Age | 0.022 | 0.009  **0.021**  0.146  0.181  0.230  0.275  0.162  0.239  0.198  0.291  0.208  0.156  0.234  **0.020**  0.333  0.007 | 0.113 | -0.041 | 0.054 | 0.351 | -0.042 | 0.050 | 0.344 |
| Educational level*^1^* | **0.138** |  | **0.002** | 0.025 | 0.112 | 0.888 | 0.022 | 0.105 | 0.899 |
| Sex | -0.118 |  | 0.401 | -0.211 | 0.887 | 0.815 | -0.221 | 0.889 | 0.798 |
| Hypertension | 0.014 |  | 0.956 | 1.752 | 1.065 | 0.106 | 1.780 | 1.063 | 0.100 |
| Diabetes | 0.265 |  | 0.250 | 0.317 | 1.367 | 0.823 | 0.336 | 1.365 | 0.815 |
| Coronary heart disease*^1^* | 0.577 |  | 0.038 | 0.748 | 1.646 | 0.654 | 0.778 | 1.651 | 0.640 |
| Cerebrovascular disease | -0.253 |  | 0.115 | -0.998 | 0.977 | 0.309 | -1.020 | 0.982 | 0.297 |
| Arrhythmia | 0.303 |  | 0.203 | -1.660 | 1.403 | 0.245 | -1.679 | 1.408 | 0.238 |
| Anti-hypertensive | -0.039 |  | 0.809 | -1.854 | 1.171 | 0.116 | -1.873 | 1.172 | 0.115 |
| Anti-diabetic | -0.074 |  | 0.788 | -1.622 | 1.739 | 0.352 | -1.558 | 1.739 | 0.372 |
| Anti-lipid agent | -0.099 |  | 0.606 | -0.451 | 1.249 | 0.719 | -0.427 | 1.247 | 0.728 |
| Anti-platelet | 0.008 |  | 0.966 | 0.207 | 0.897 | 0.824 | 0.159 | 0.895 | 0.867 |
| Anti-coagulant*^2,3^* | -0.361 |  | 0.116 | 2.985 | 1.396 | 0.040 | 2.970 | 1.389 | 0.040 |
| Frailty*^1^* | **-0.114** |  | **0.003** | 0.025 | 0.067 | 0.784 | 0.020 | 0.066 | 0.812 |
| Follow-up duration | 0.029 |  | 0.623 | -0.023 | 0.219 | 0.555 | 0.009 | 0.059 | 0.825 |
| Triglyceride | 0.006 |  | 0.778 | 0.005 | 0.013 | 0.399 | 0.001 | 0.017 | 0.443 |

**Supplementary Table 6. (Continued)**

|  | Model 1 | | | Model 2 | | | Model 3 | | |
| --- | --- | --- | --- | --- | --- | --- | --- | --- | --- |
|  | *b* | SE | *p*-value | *b* | SE | *p*-value | *b* | SE | *p*-value |
| ***Dementia-D*** |  |  |  |  |  |  |  |  |  |
| Age | -0.009 | 0.011 | 0.419 | 0.054 | 0.073 | 0.462 | 0.056 | 0.073 | 0.438 |
| Educational level*^1^* | **0.126** | **0.020** | **0.000** | -0.055 | 0.135 | 0.684 | -0.051 | 0.135 | 0.703 |
| Sex | 0.051 | 0.171 | 0.765 | 0.761 | 1.114 | 0.495 | 0.748 | 1.113 | 0.502 |
| Hypertension | 0.277 | 0.205 | 0.177 | 1.603 | 1.337 | 0.231 | 1.615 | 1.336 | 0.227 |
| Diabetes*^2,3^* | 0.309 | 0.287 | 0.282 | **-8.068** | **1.874** | **0.000** | **-8.063** | **1.872** | **0.000** |
| Coronary heart disease | 0.249 | 0.310 | 0.422 | -1.522 | 2.022 | 0.452 | -1.667 | 2.020 | 0.409 |
| Cerebrovascular disease*^2,3^* | -0.118 | 0.192 | 0.541 | **2.921** | **1.248** | **0.020** | **2.759** | **1.251** | **0.028** |
| Arrhythmia | -0.198 | 0.319 | 0.534 | 2.439 | 2.084 | 0.242 | 2.598 | 2.084 | 0.213 |
| Anti-hypertensive | 0.137 | 0.216 | 0.527 | -1.434 | 1.412 | 0.310 | -1.380 | 1.412 | 0.329 |
| Anti-diabetic*^2,3^* | -0.426 | 0.349 | 0.222 | **6.247** | **2.279** | **0.006** | **6.276** | **2.272** | **0.006** |
| Anti-lipid agent | -0.130 | 0.233 | 0.578 | -0.681 | 1.524 | 0.655 | -0.519 | 1.513 | 0.732 |
| Anti-platelet | -0.101 | 0.171 | 0.553 | -0.814 | 1.117 | 0.466 | -0.732 | 1.116 | 0.512 |
| Anti-coagulant | 0.008 | 0.266 | 0.976 | 0.644 | 1.735 | 0.711 | 0.689 | 1.732 | 0.691 |
| Frailty*^1^* | **-0.070** | **0.012** | **0.000** | 0.005 | 0.079 | 0.950 | 0.001 | 0.079 | 0.985 |
| Follow-up duration | 0.024 | 0.337 | 0.624 | -0.022 | 0.215 | 0.560 | 0.005 | 0.053 | 0.832 |
| Triglyceride*^1^* | **0.002** | **0.001** | **0.045** | 0.008 | 0.006 | 0.185 | -0.022 | 0.013 | 0.098 |

Notes and abbreviations are the same as those used in supplementary Tables 2 and 3.

**Supplementary Figure 1. Relationships between HDL-c and Cognitive Functions**


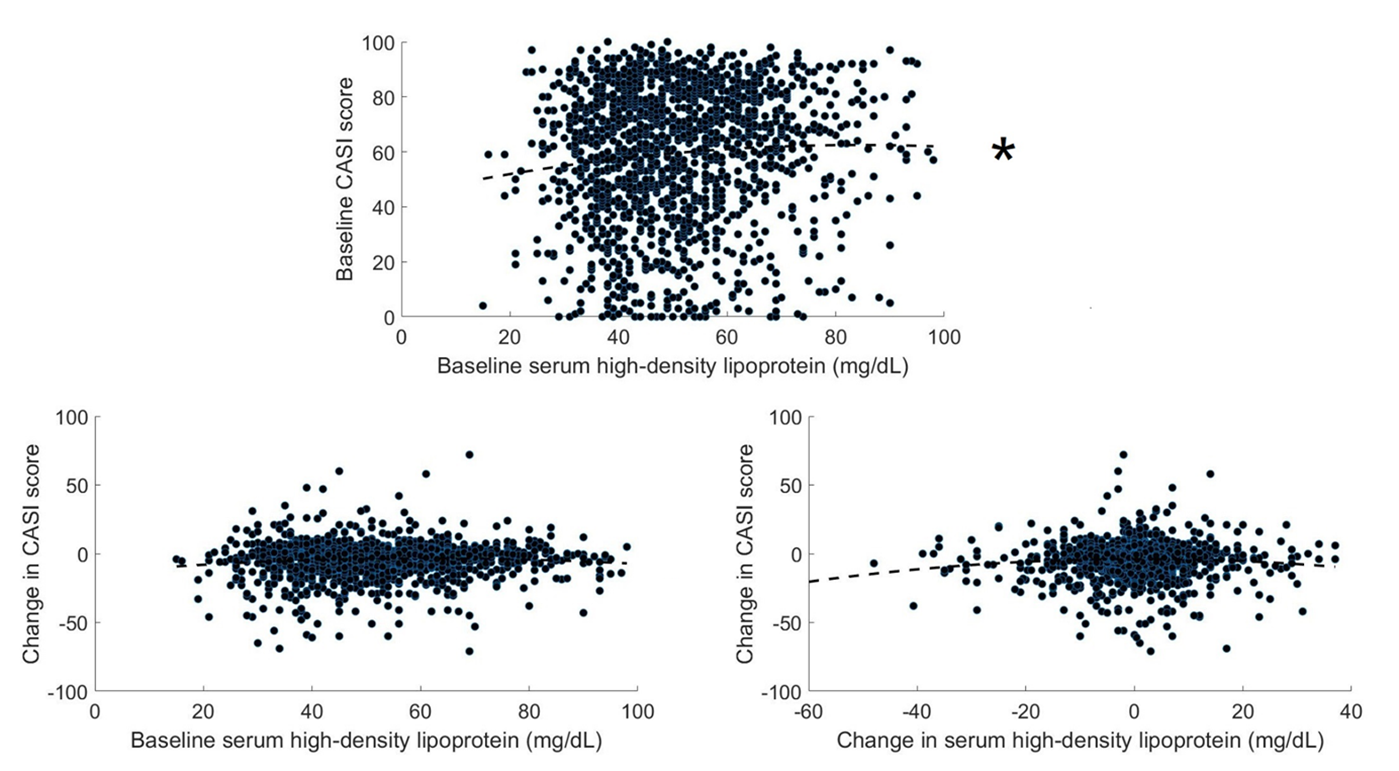


*Note.* The upper panel denotes the relationship between baseline cholesterol levels and baseline CASI scores. The lower left plot denotes the relationship between baseline cholesterol levels and changes in CASI scores. The lower right plot denotes the relationship between changes in cholesterol levels and CASI scores. *Abbreviations.* CASI: Cognitive Assessment Screening Instrument; HDL-c: High-density lipoprotein cholesterol. Asterisk sign denotes a significant quadratic relationship.

**Supplementary Figure 2. Relationships between TG and Cognitive Functions**

**
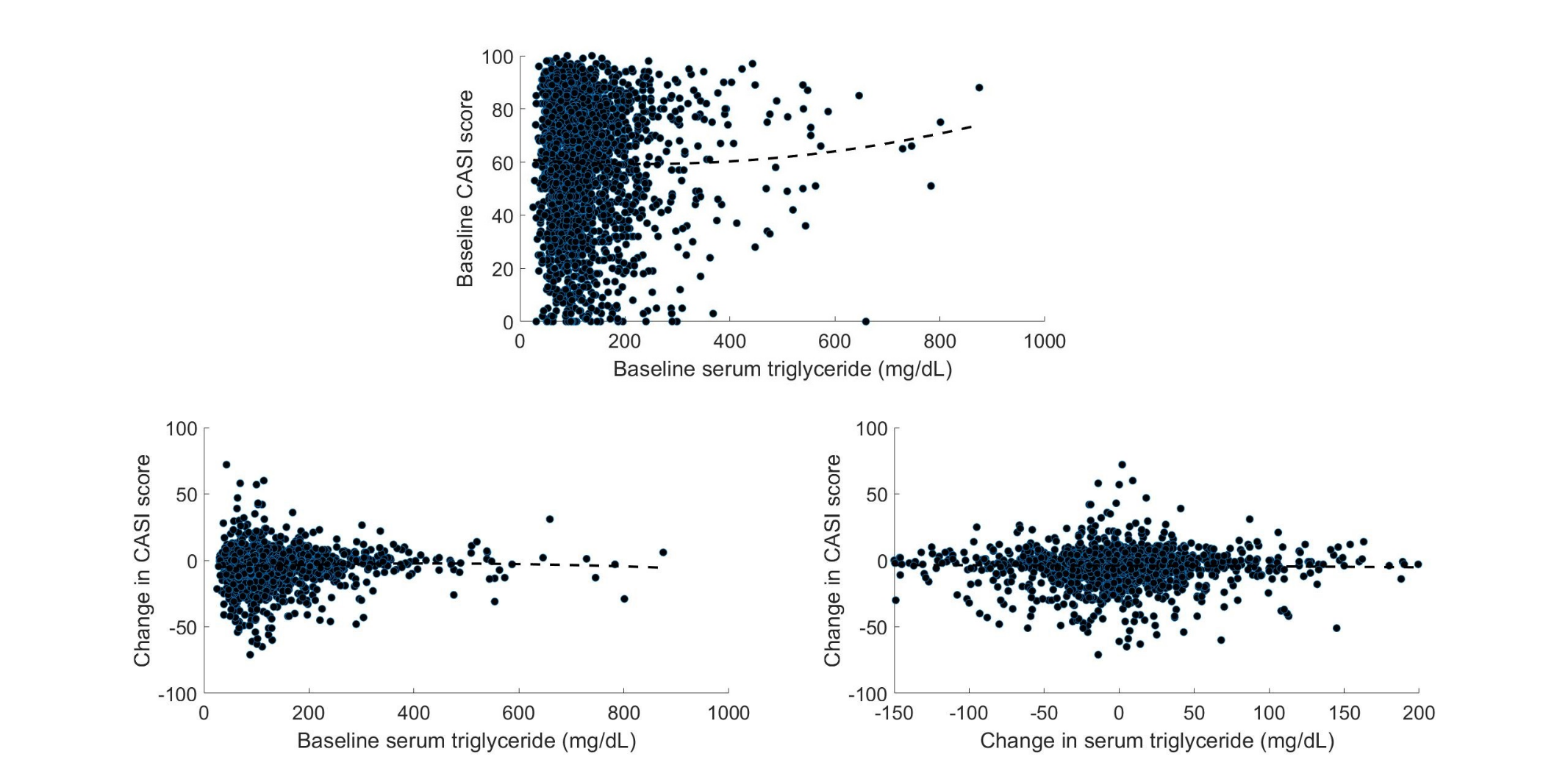
**

*Abbreviations.* TG: Triglyceride. Other Notes and Abbreviations are the same as those used in supplementary Figure 1. Other notes and abbreviations are the same as those used in supplementary Figure 1.

**Supplementary Figure 3. Relationships between TC and Cognitive Function across Individuals with Different Cognitive States**


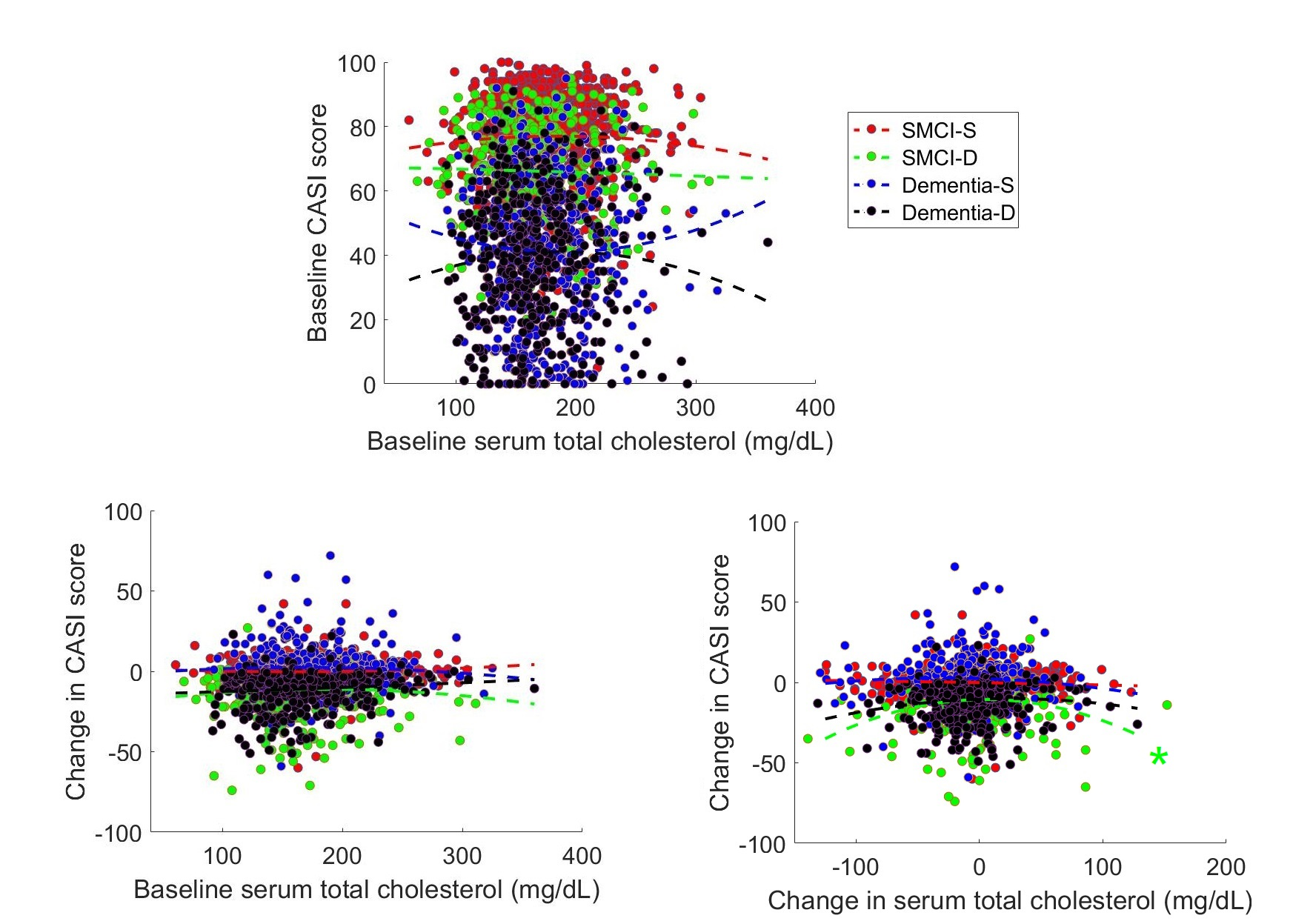


Notes and Abbreviations are the same as those used in supplementary Figure 1.

**Supplementary Figure 4. Relationships between TG and Cognitive Function across Individuals with Different Cognitive States**

**
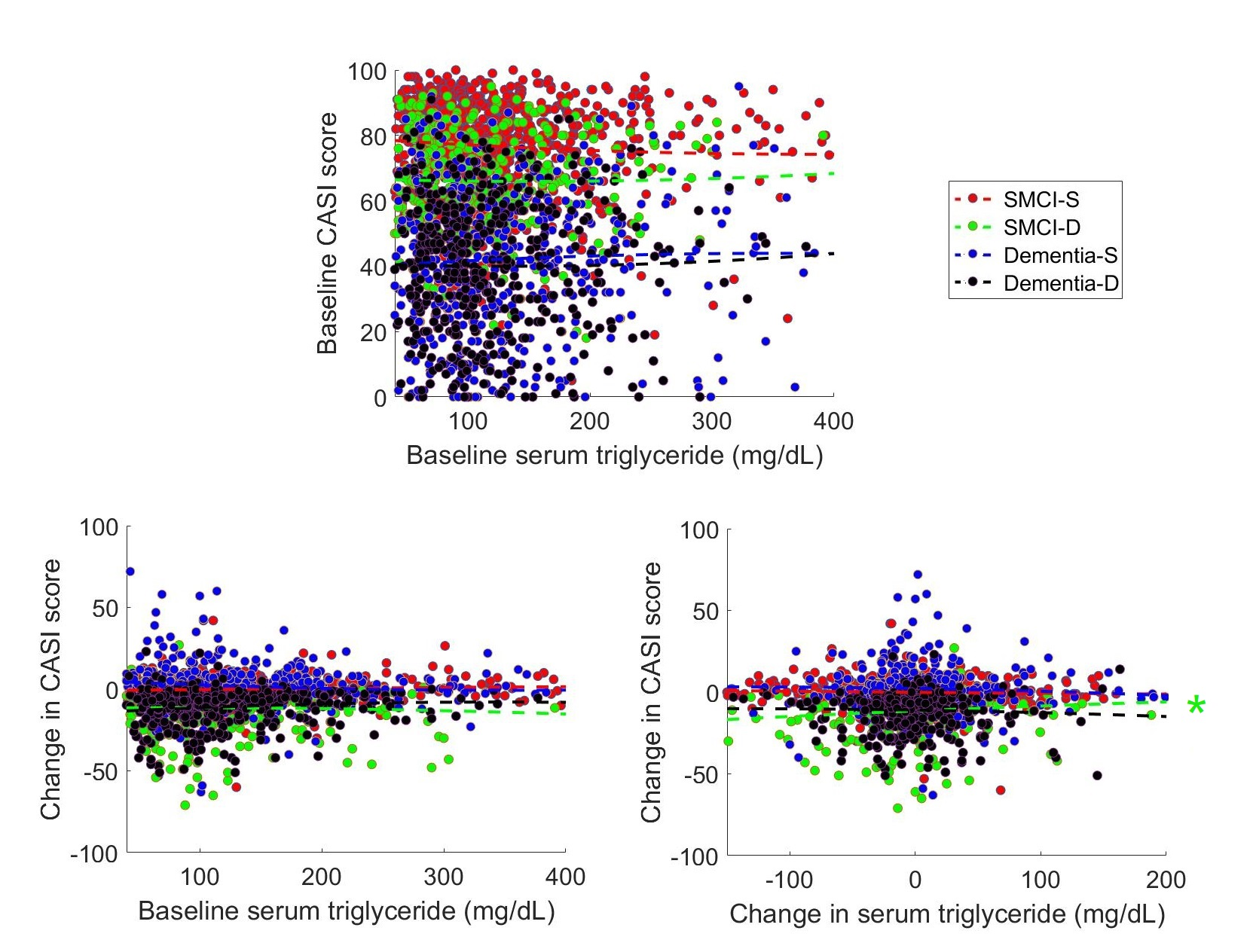
**

Note and abbreviations are the same as those used in supplementary Figure 1.

**Supplementary Figure 5. Relationships between HDL-c and Cognitive Functions across Individuals with Different Cognitive States.**

**
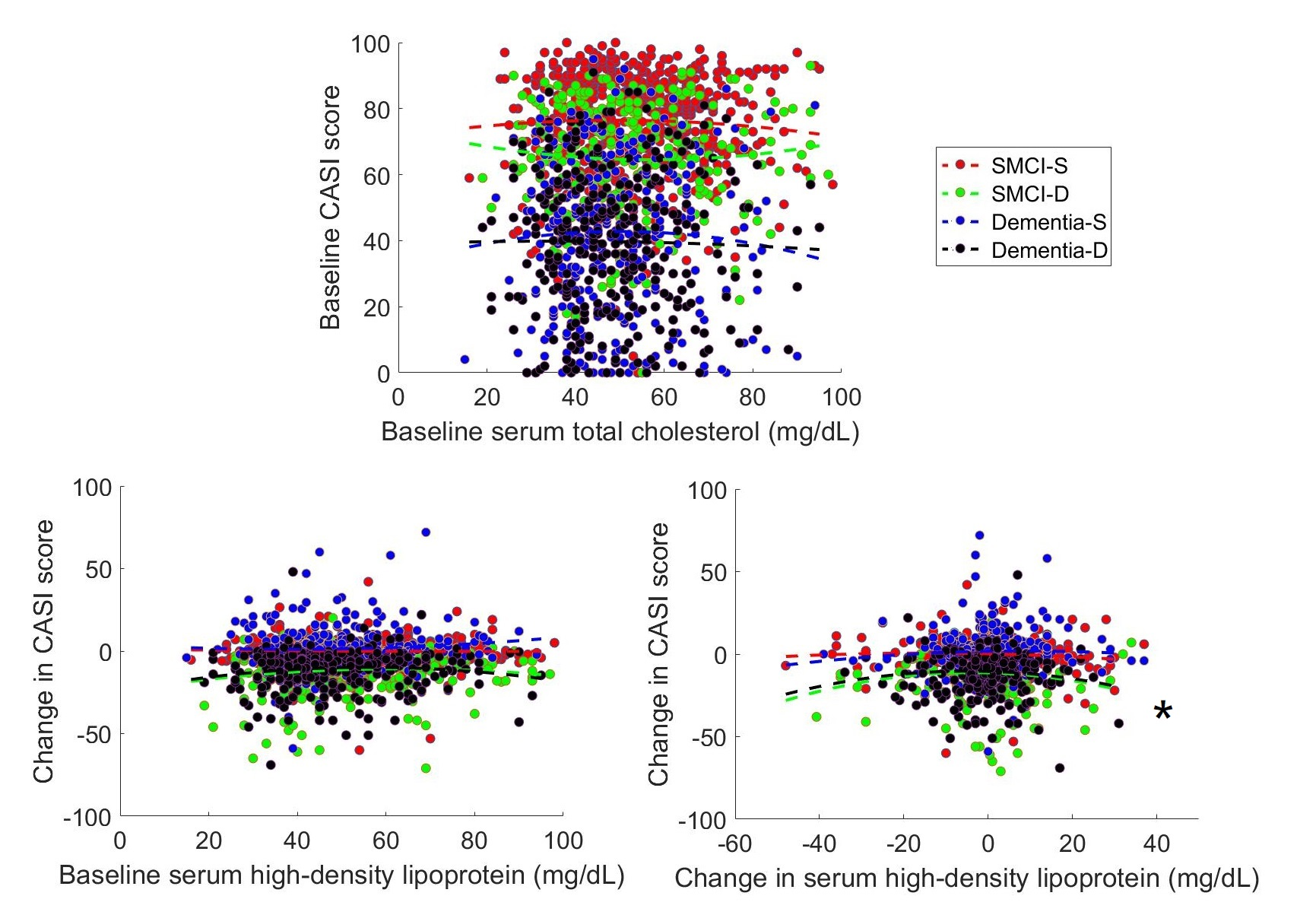
**

Notes and abbreviations are the same as those used in supplementary Figure 1.

**Supplementary Figure 6. Comparisons of Cognitive Changes among Individuals with Drastic Changes in Serum Cholesterol.**
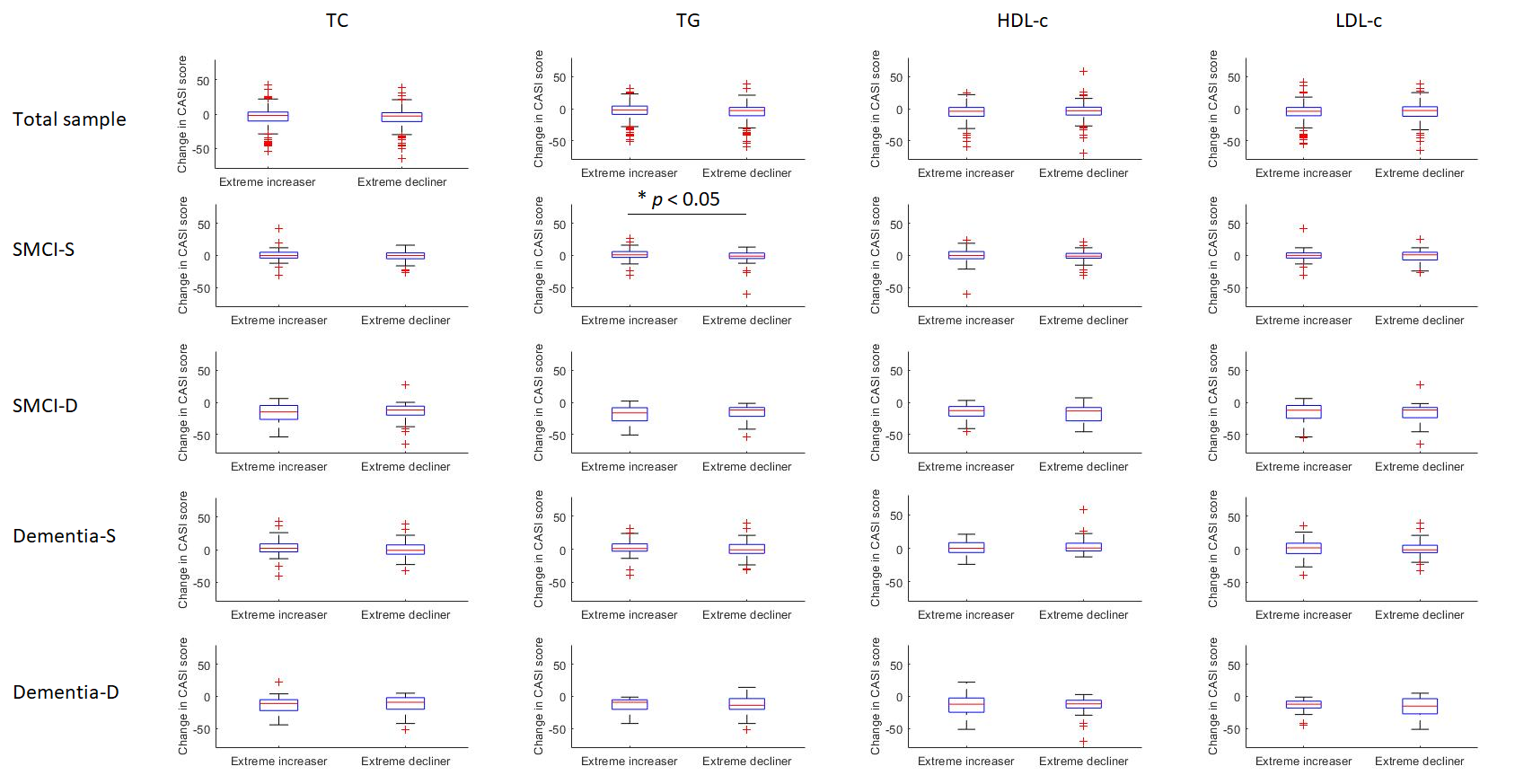


*Note.* Red cross denotes outlier whose value is more than 1.5 times the interquartile range; box denotes for interquartile range with the horizontal red line as median.
